# Geometry and Material Criteria for Low-Carbon Design of I/H-Beams in Sustainable Steel Structures Considering Both Mechanical Properties and Carbon Emissions

**DOI:** 10.3390/ma18214930

**Published:** 2025-10-28

**Authors:** Jitao Bai, Keyong Yang, Zhonghao Chen, Jiahe Liang, Simiao Zhang, Yu Diao

**Affiliations:** 1 Department of Civil Engineering, Tianjin University, Tianjin 300072, China; jitaobai_123@tju.edu.cn; 2 Key Laboratory of Coast Civil Structures and Safety of Ministry of Education, Tianjin University, Tianjin 300072, China; 3 College of Intelligence and Computing, Tianjin University, Tianjin 300072, China; ykyong@tju.edu.cn (K.Y.); diogeneschen@tju.edu.cn (Z.C.); 4 Faculty of Architecture, KU Leuven, 9000 Gent, Belgium; liangjiahe117@gmail.com; 5 Software Engineering Institute, East China Normal University, Shanghai 200062, China; smzhang@stu.ecnu.edu.cn; 6 National Science Center for Earthquake Engineering, Tianjin University, Tianjin 300350, China; 7 State Key Laboratory of Hydraulic Engineering Intelligent Construction and Operation, Tianjin University, Tianjin 300350, China

**Keywords:** design criteria, I/H-beams, carbon emissions, mechanical properties, construction steel

## Abstract

Construction steel is responsible for considerable amounts of carbon emissions in building sectors, and promoting the low-carbon design of steel components is conducive to the sustainable development of the industry. As one of the most typical components, I/H-beams are widely used in steel structures. In this paper, a new comprehensive index named carbon-capacity ratio (CCR) was proposed considering both mechanical properties and carbon emissions of I/H-beams, based on which the geometry coefficient and material coefficient were derived. Quantitative investigation was then conducted on the geometry coefficient to figure out the effects of different geometry variables, and the geometry criteria for low-carbon design of steel beams were concluded considering different load conditions. Results show that for double-symmetric cross-sections bearing flexural loads, larger flange width and beam height are suggested, while for single-symmetric cross-sections bearing flexural loads, increasing beam height as well as flange width and thickness can all contribute to sustainable beam designs, but adopting large beam height is the most effective. For cross-sections bearing shear loads, increasing beam height and web thickness would be beneficial. The feasible design domain (FDD) for geometry variables was proposed to be predicted with either linear or hyperbolic criteria depending on different loads and cross-sections. Additionally, a qualitative discussion was also given on the material coefficient, and steel with higher strength or that produced from recycled scrap using energy-saving technologies, as well as new prototyping techniques with lower energy and material loss, are recommended for beam fabrication. This study is expected to serve as a preliminary supplement to the blank in current codes or standards for low-carbon design of construction steel.

## 1. Introduction

Steel structures have been increasingly adopted in high-rise high-density modern buildings [1]. Compared with reinforced concrete solid slab systems or composite structural systems, steel structures are superior in terms of lightweight and fast construction, but in the meanwhile, have the highest environmental impact [2]. One aspect for the environmental impact of steel structures comes from their global warming potential [3], which is mainly induced by extensive carbon emissions [4]. Studies have found that among all factors that can contribute to the carbon emissions of steel structures, steel is actually the biggest culprit [5,6]. Each year, manufacturing steel for construction generates about 3.5% of global carbon emissions [7]. Since the building sector is the main source for global carbon emissions [8,9], promoting the low-carbon design of steel structures is conducive to the sustainable development of the construction industry. As a response, various alternatives have been proposed, such as combining steel with specially designed concrete [10] or renewable materials like timber [11,12,13,14,15,16] and bamboo [17] to form composite components, rehabilitating damaged steel components to extend the service life [18], and promoting the reuse of the recycled steel [19,20,21]. But what should be acknowledged is that there is still a great potential for carbon reduction just from the optimization of construction steel itself.

Optimization of construction steel can be conducted on either the structure or element level. Structure-level optimization focuses on the layout of the components in steel structures so that structure forms with lower global warming potential (e.g., double-beam system) can be determined [22,23]. Features of the components may also be involved, but mostly the cross-sectional area, in which the minimum cross-sections are selected for each component in the given structure from a prescribed list of standard cross-sections considering their specific loading conditions or design requirements [24,25,26,27], finally leading to the maximum steel savings of the whole structure. Strictly speaking, what these studies optimize are more like the combinations of standard cross-sections instead of the design of the cross-section itself, as tailored optimization of each component for non-standard cross-sections is time-consuming and can take lots of computation for structures with thousands of components and is thus only applicable for some simple structures like portal frames [28] or trusses [29]. Structure-level optimization has been proven to be effective in lowering the carbon emissions of the whole structure. However, since the forms of different structures can be varying, such optimization is only case-to-case, and the conclusions drawn from one case can hardly be generalized and applied to other cases. Moreover, structure-level optimization is performed on existing structures, meaning that it can be performed only when the initial design is finished. That has caused another problem: changing the completed design requires much more work than making changes at the start.

Element-level optimization focuses on the properties of an individual component like a beam or column, or more exactly, the shape and dimension of the cross-section, as the performance of a steel component is generally dominated by its cross-section. Compared with structure-level optimization, element-level optimization runs in a more basic level and is expected to provide more sustainable choices at the start of the initial design [30]. It was found in steel structures that among all types of components, beams have the biggest impact on carbon emissions of the whole structure throughout the life cycle [2,31]. In this case, it is promising to lower the carbon emissions of steel structures through the optimization of steel beams. Current studies on steel beam optimization mainly focus on their mechanical properties such as flexural capacity [32,33,34,35,36], buckling resistance [37,38], as well as the behavior in response to coupled loading [39,40]. Performance under some special conditions like seismic performance [41] and resistance to ground stress [42] was also considered. But in general, environmental impact is rarely treated as the objective of steel beam optimization, despite very limited literature on embodied energy [43], thermal performance [44], and carbon emissions [45]. Even though some studies have considered minimum material use while optimizing the mechanical properties [46,47,48,49,50,51], they are still faced with the problem of generalization, as just like the structure-level optimization, the loading conditions under which the optimization was performed can vary from one to another. Another problem comes from the methods involved in the optimization, mostly machine learning methods (e.g., reinforcement learning (RL) and back-propagation (BP) neural network) [36] and stochastic algorithms like meta-heuristic algorithms (e.g., genetic algorithm (GA) and particle swarm optimization (PSO) algorithm) [32,38,39,40,42,48,49,51]. Due to the inherent black-box property of these methods, the effects of design variables on optimization objectives remain unclear. While that is indeed of significance, since the optimal results obtained are not always applicable for all engineering scenarios, even those with the same loading conditions, and knowing how design variables can actually lead to the optimum or near optimum is more helpful for low-carbon structure design with higher flexibility. A few studies have conducted variable analysis [52,53,54], but unfortunately, they are mostly mechanical-related and with still a gap towards low-carbon requirement.

Some previous studies have indeed proposed more generalized methods concerning element-level optimization that take both the mechanical properties and environmental impacts into account and enable the analysis on individual design variables. For example, Ashby [55] described the performance of components (e.g., the combined mechanical and environmental performance) as the aggregate of three indices, i.e., the functional index, geometric index, and material index. Normally, these indices are independent of each other, though sometimes geometry variables [55,56,57] or load parameters [58] could be integrated into the material indices, depending on the design goals or load conditions considered. That has brought huge simplification to component optimization, as the decomposition of component performance into independent indices means the optimal performance can be simply achieved by optimizing each index individually (e.g., the optimal material choice can be easily determined through material property charts without considering the geometry or functionality parameters of components [55,59,60,61]), and when used as constraints, there is no need to treat these indices simultaneously. Despite the great advancement, however, special manipulation like the construction of penalty function [55,60,62] or value function [59] is still required in optimization, and either graphical or analytical methods [55] will be used to resolve the indices as constraints, leaving room for further reduction in optimization complexity. Additionally, for indices serving as constraints, specific design requirements on the variables involved, such as the bearing capacity and dimensions, must be known [55,63,64]. That arouses the idea for constraints that always hold regardless of the design requirements.

**Figure 1:**
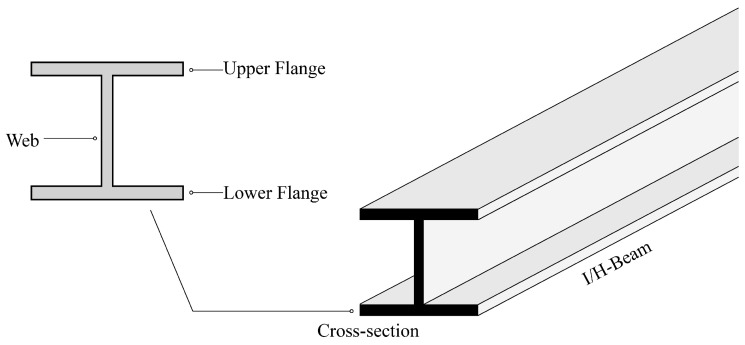
Schematic diagram for I/H-beam and its cross-section.

I/H-beams (Figure 1) are one of the most widely used beam types in steel structures. In this paper, a new comprehensive index (carbon-capacity ratio, CCR) measuring both mechanical properties and carbon emissions of I/H-beams was proposed, based on which the geometry coefficient and material coefficient were derived. Quantitative investigation was then conducted on the geometry coefficient to figure out the effects of different geometry variables, and the geometry criteria for low-carbon design of steel beams were concluded considering different load conditions. Moreover, a qualitative discussion was also given on the material coefficient to give some suggestions on material selection and beam fabrication. This study offers the following contributions:

- The proposed CCR integrates both mechanical properties and carbon emissions and transforms the comprehensive performance into the geometry coefficient and material coefficient, which are mutually independent and allows for simpler performance optimization of components through the separate optimization of the two indices.
- The geometry coefficient was numerically investigated as an intermediate variable to establish the geometry criteria for the low-carbon design of I/H-beams, including the effect of different geometry variables as well as the feasible design domain (FDD) of I/H-beams under different load conditions. The FDD is a more generalized and universal analytical design recommendation that has nothing to do with predetermined design requirements and can be directly used as the constraints that are easier to resolve in the low-carbon optimization of I/H-beams.
- The material coefficient allows the life-cycle carbon emissions to be taken into account, which was analyzed to give the material criteria on material selection and beam fabrication regarding the low-carbon design of I/H-beams. When used as the constraints in optimization, there is no need to deal with the complex connections between the material coefficient and the FDD, thus making the optimization more efficient.

## 2. Methodology

### 2.1. Mechanical Properties

Mechanical properties of components must be given priority in structure design to ensure safety. In this study, two mechanical indexes were examined, including the flexural capacity and ultimate shear strength of I/H-beams, as they are the most typical load conditions for beams. The steel I/H-beams were assumed to be perfectly elastic, and the post-buckling capacity was not considered. Both the flexural capacity and ultimate shear strength were calculated based on mechanics of materials rather than the code or standard of a specific country or society so that the findings of the study are broad-spectrum and compatible with all current codes or standards for steel structures.

#### 2.1.1. Flexural Capacity

According to the mechanics of materials, the flexural capacity ($M_{u}$) of steel beams in elastic state can be calculated as Equation (1), where W is the full section modulus of the beam, and f is the design value of tensile strength for steel used in the beam.

(1) $$M_{u}=Wf$$

The design value of tensile strength f is a constant for a specific type of steel, while for the full section modulus W, it is related to the cross-sections of beams. Based on the geometry features, the cross-sections of I/H-beams can be classified as double-symmetric and single-symmetric, and the flexural capacity is discussed accordingly as follows.

##### Double-Symmetric Cross-Section

**Figure 2:**
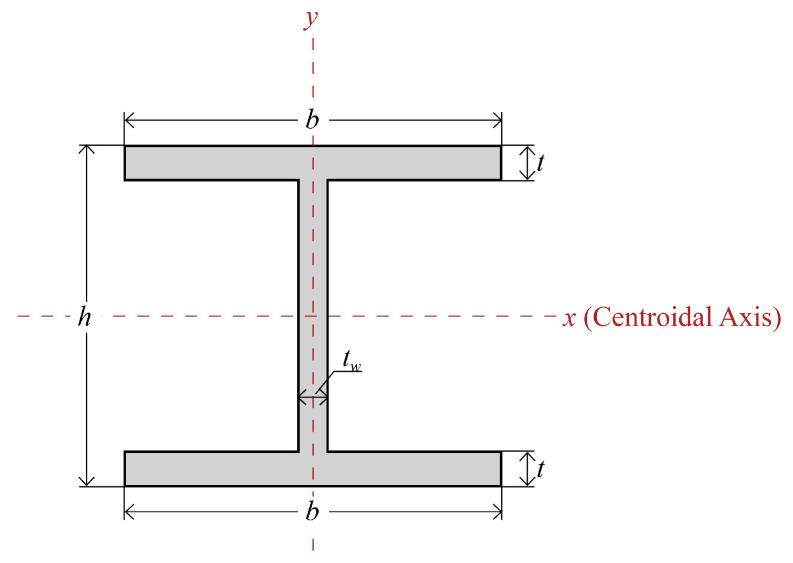
Double-symmetric cross-section.

In double-symmetric cross-sections, the upper and lower flange are of the same size, as illustrated in Figure 2. In this case, the centroidal axis goes along the horizontal axis of symmetry (line *x*), and the full section modulus W can be expressed as Equation (2), where h is the height of the beam, and I is the moment of inertia calculated as Equation (3) with the width of the flange, the thickness of the flange, and the thickness of the web denoted by b, t, and $t_{w}$, respectively.

(2) $$W=\frac{2I}{h}$$

(3) $$I=\frac{1}{6}bt^{3}+\frac{1}{2}bt{\left(h-t\right)}^{2}+\frac{2}{3}t_{w}{\left(\frac{h}{2}-t\right)}^{3}$$

##### Single-Symmetric Cross-Section

**Figure 3:**
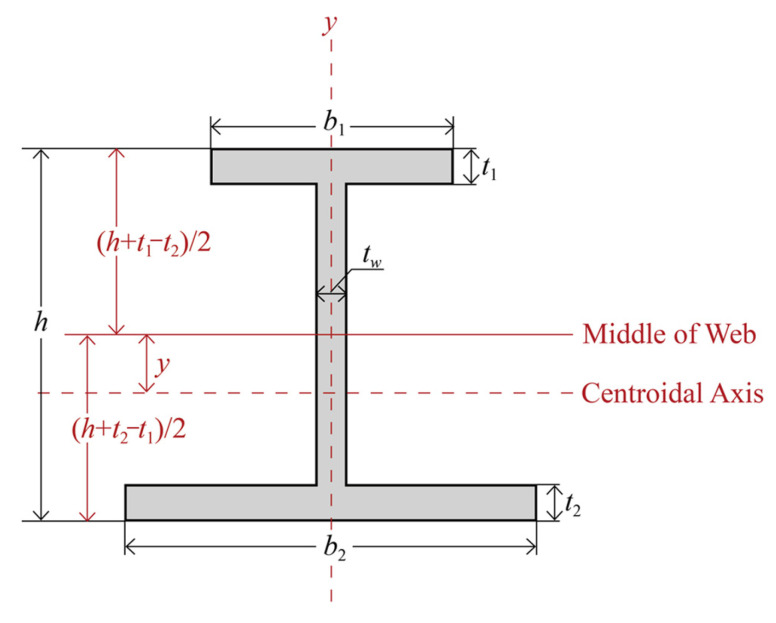
Single-symmetric cross-section.

The upper and lower flange in single-symmetric cross-sections have different dimensions, as shown in Figure 3. Although it is not as regular as double-symmetric cross-sections, such heterogeneous configuration is believed to have greater potential in terms of sustainability [65]. Compared with the case for double-symmetric cross-section, the single-symmetric geometry has caused a dislocation of the centroidal axis, which will no longer go through the middle of the beam height. Instead, a distance of y is generated between the centroidal axis and the middle of the web height, as illustrated in Figure 3. Such dislocation can be calculated according to Equation (4), in which $b_{1}$ is the width of the upper (usually compressive) flange, $t_{1}$ is the thickness of the upper flange, $b_{2}$ is the width of the lower (usually tensile) flange, $t_{2}$ is the thickness of the lower flange, and A is the total area of the cross-section expressed as Equation (5). Symbol h and $t_{w}$ still represent the height of the beam and the thickness of the web, respectively. Note that the dislocation y can be negative in value, which means the centroidal axis is above the middle of the web height, and vice versa.

(4) $$y=\frac{b_{2}t_{2}\left(h-t_{1}\right)-b_{1}t_{1}\left(h-t_{2}\right)}{2A}$$

(5) $$A=b_{1}t_{1}+b_{2}t_{2}+t_{w}\left(h-t_{1}-t_{2}\right)$$

With the dislocation y obtained, the distance between the centroidal axis and the top and bottom of the beam, denoted by $y_{1}$ and $y_{2}$, respectively, can be simply expressed as Equations (6) and (7), and the moment of inertia (I) for single-symmetric cross-section is readdressed as Equation (8).

(6) $$y_{1}=\frac{h+t_{1}-t_{2}}{2}+y$$

(7) $$y_{2}=\frac{h+t_{2}-t_{1}}{2}-y$$

(8) $$I=b_{1}t_{1}{\left(y_{1}-\frac{t_{1}}{2}\right)}^{2}+b_{2}t_{2}{\left(y_{2}-\frac{t_{2}}{2}\right)}^{2}+\frac{1}{3}t_{w}\left[{\left(y_{1}-t_{1}\right)}^{3}+{\left(y_{2}-t_{2}\right)}^{3}\right]+\frac{1}{12}\left(b_{1}{t_{1}}^{3}+b_{2}{t_{2}}^{3}\right)$$

Then the section modulus, including the section modulus for compressive flange ($W_{1}$) and that for tensile flange ($W_{2}$), can be calculated as follows:

(9) $$W_{1}=\frac{I}{y_{1}}$$

(10) $$W_{2}=\frac{I}{y_{2}}$$

According to Equation (1), two section moduli will bring two different values for flexural capacity, and obviously the actual flexural capacity adopts the smaller one as it is dominated by the weaker flange. That means there is still a strength margin in the flange with larger section modulus and the steel of this part is not fully utilized as expected. In other words, there is still room for steel savings, which represents lower carbon emissions. In this case, a constraint as Equation (11) will be required to guarantee the full use of the material.

(11) $$W_{1}=W_{2}$$

Substitute Equations (4)–(10) into Equation (11), and the constraint for geometry variables can be obtained as Equation (12). Actually, such constraint has suggested two different cases:

(12) $$b_{1}t_{1}\left(h-t_{1}\right)-b_{2}t_{2}\left(h-t_{2}\right)+t_{w}\left(t_{2}-t_{1}\right)\left(h-t_{1}-t_{2}\right)=0$$

(a) If there is $t_{1}=t_{2}$, then the relation of $b_{1}=b_{2}$ can be derived from Equation (12). That is, the cross-section has reduced to be double-symmetric with only four independent variables including the width (b) and thickness (t) of the flange, the height of the beam (h), as well as the thickness of the web ($t_{w}$), as previously discussed.
(b) If there is $t_{1}\neq t_{2}$, then the relation as Equation (13) can be derived. The cross-section is still single-symmetric, but the number of independent variables has reduced from 6 to 5, including the width ($b_{1}$) and thickness ($t_{1}$) of the upper flange, the width ($b_{2}$) and thickness ($t_{2}$) of the lower flange, as well as the height of the beam (h).
  (13) $$t_{w}=\left|\frac{b_{1}t_{1}\left(h-t_{1}\right)-b_{2}t_{2}\left(h-t_{2}\right)}{\left(t_{1}-t_{2}\right)\left(h-t_{1}-t_{2}\right)}\right|$$

#### 2.1.2. Ultimate Shear Strength

The ultimate shear strength ($V_{u}$) of I/H-beams can be calculated according to Equation (14), where S is the static (area) moment of the gross section above (or below) the neutral axis to the neutral axis, and $f_{v}$ is the design value of shear strength for steel used in the beam. For I/H-beams in elastic state, the neutral axis overlaps with the centroidal axis, and the static moment S can thereby be calculated as Equation (15). While the moment of inertia I is calculated either by Equation (3) or Equation (8) depending on the symmetry of the cross-section.

(14) $$V_{u}=\frac{I}{S}t_{w}f_{v}$$

(15) $$S=b_{1}t_{1}\left(y_{1}-\frac{t_{1}}{2}\right)+\frac{1}{2}t_{w}{\left(y_{1}-t_{1}\right)}^{2}$$

### 2.2. Carbon Emissions

The total carbon emissions for one steel beam ($C_{bs}$) can be simply expressed as Equation (16), where $C_{s}$ is the carbon emissions generated per unit volume of steel, and l is the span of the beam.

(16) $$C_{bs}=AlC_{s}$$

Definitely, the total carbon emissions of a beam are related to its span. However, the span is only determined by the demand in practical engineering and cannot be prescribed. As a solution, the carbon emissions for cross-section ($C_{s0}$) are defined herein as Equation (17), which has eliminated the influence of beam span.

(17) $$C_{s0}=\frac{C_{bs}}{l}=AC_{s}$$

### 2.3. Comprehensive Index

In order to integrate the mechanical properties into low-carbon design, a comprehensive index named carbon-capacity ratio (CCR) was proposed in this study as $R_{c}$ in Equation (18), which is the ratio of the carbon emissions for cross-section ($C_{s0}$) to the capacity of beam cross-section (F).

(18) $$R_{c}=\frac{C_{s0}}{F}$$

As for flexural capacity, the CCR can be obtained as Equation (19). While for ultimate shear strength, the CCR can be expressed as Equation (20). Obviously, the CCR is expected to be lower as it means the same capacity can be achieved with lower carbon emissions.

(19) $$R_{csM}=\frac{C_{s0}}{M_{u}}=\frac{AC_{s}}{Wf}$$

(20) $$R_{csV}=\frac{C_{s0}}{V_{u}}=\frac{ASC_{s}}{It_{w}f_{v}}$$

The proposed index CCR can be decomposed as the product of the geometry coefficient ($\varphi_{g}$) and material coefficient ($\gamma_{m}$), as shown in Equation (21). For CCR considering flexural capacity, the geometry coefficient and material coefficient are given in Equation (22), while for that considering ultimate shear strength, the two terms are given in Equation (23). It can be found that the geometry coefficient is only related to the geometry features of beam cross-sections and has nothing to do with the material, while the material coefficient is related to the properties of the material itself, including the strength properties as well as the carbon emissions in material production. What should be clarified is that the geometry coefficient given herein is never the shape factor defined in previous studies [55,56,57]. In fact, they are different in both the definition and the formulae: the geometry coefficient was derived by extracting geometry-related variables from the comprehensive index CCR, while the shape factor was defined as the ratio of the stiffness or strength of the shaped section to that of a “neutral” reference shape (e.g., that of a solid square section with the same cross-sectional area) [56]. Such difference is clearly evidenced by the units of the indices: the proposed geometry coefficient in this study can sometimes have its own unit, depending on the load conditions (e.g., mm^−1^ for $\varphi_{gM}$), while the shape factor in previous studies is always dimensionless, as it was developed to separate section-area from section-shape, i.e., components with the same shape factor, though they could differ in size, have cross-sections in the same shape [57]. By consolidating all the geometry variables rather than merely those directly related to the cross-section shape, the geometry coefficient has facilitated the understanding about the effects of different geometry variables on the comprehensive component performance instead of just how they contribute to the component shape. And further, it paves the way for the establishment of the constraint sets that can be more efficiently used in component performance optimization, i.e., the feasible design domain that will be elaborated in Section 3.

(21) $$R_{c}=\varphi_{g}\gamma_{m}$$

(22) $$\left\{\begin{array}{c} \varphi_{gM}=\frac{A}{W}\\ \gamma_{mM}=\frac{C_{s}}{f} \end{array}\right.$$

(23) $$\left\{\begin{array}{c} \varphi_{gV}=\frac{AS}{It_{w}}\\ \gamma_{mV}=\frac{C_{s}}{f_{v}} \end{array}\right.$$

In this study, quantitative investigation was conducted on the geometry coefficient to figure out the effects of different geometry variables, based on which the geometry criteria for low-carbon design of steel beams were concluded considering different load conditions. In addition, a qualitative discussion was also given on the material coefficient to give some suggestions on material selection and beam manufacture. All design variables involved in the discussion of Section 3 follow the ranges normally used in China-made I/H-beams [66], as given in Table 1.

## 3. Results and Discussion

### 3.1. Geometry Criteria

#### 3.1.1. Criteria Governed by Flexural Capacity

As discussed in Section 2.1.1, the calculation of flexural capacity is related to the symmetry of beam cross-section. Hence in this section, the geometry criteria were respectively discussed following different symmetric features.

##### Double-Symmetric Cross-Section

**Figure 4:**
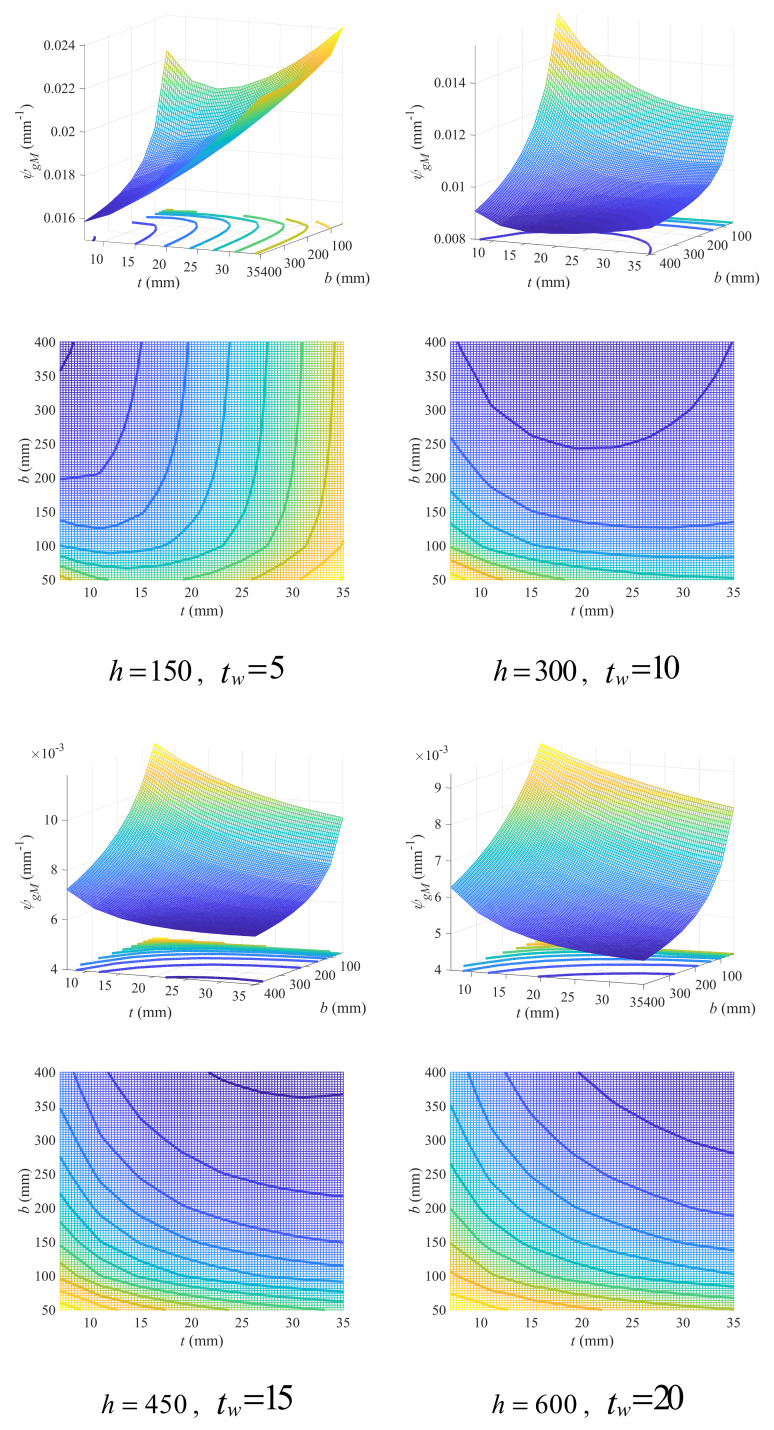
Effects of flange width and thickness on geometry coefficient under different web thickness and beam height (mm).

In double-symmetric cross-sections, there are four independent variables, including two concerning flange (i.e., flange width b and thickness t) and the other two concerning web (web thickness $t_{w}$) and beam height (h). The variation in the geometry coefficient ($\varphi_{gM}$) with flange variables under different beam height and web thickness has been plotted in Figure 4 (due to limited space, please refer to Figure S1 in Supplementary Material for full figures). It can be found that the increase in web thickness will also lead to an increase in the geometry coefficient, while the increase in beam height will on the contrary lead to a decrease in the geometry coefficient. Since a small geometry coefficient is conducive to lower CCR, which means a design with lower carbon emissions, higher cross-sections with thinner webs are suggested.

Similarly to beam height, the increase in flange width will also lower the geometry coefficient, while the effects of flange thickness are complex and related to all three other variables. For a large flange width, the geometry coefficient increased with the increase in flange thickness at lower beam height (*h* = 150 mm). However, such increase was alleviated as the web thickness increased. With the increase in beam height, for example, at the beam height of 300 mm, the increase in the geometry coefficient with flange thickness can only be observed at small web thickness, which soon transformed to decrease as the web thickness increased. For beam heights greater than 300 mm, the geometry coefficient decreased with the increase in flange thickness, regardless of web thickness. Similar phenomena were also found in cases with small flange width: at a small beam height (*h* = 150 mm), the geometry coefficient first decreased and then increased with the increase in flange thickness, but such increase in trend was alleviated as the web thickness increased. With the increase in beam height, the geometry coefficient transformed to monotonically decreasing with flange thickness. The difference lies in the effects of web thickness, which can promote the decrease in the geometry coefficient with flange thickness at larger flange width, while inhibiting that decrease at smaller flange width.

The variation in the geometry coefficient with flange variables can be more intuitively observed through contour graphs in Figure 4 (see Figure S1 in Supplementary Material for full figures), where a darker cold color indicates smaller value of the geometry coefficient and a transformation to warm color indicates the increase in the geometry coefficient. A region in contour graph with dark cold color and sparse contours means a gentle variation in the geometry coefficient at lower values, and thereby is more suitable to adopt in beam design. In this sense, such a region is herein defined as feasible design domain (FDD). It can be found that at lower beam height (*h* = 150 mm), the FDD occurred at small flange thickness with large flange width, and with the increase in web thickness, the FDD got larger. However, the case is just the opposite when the beam got higher (*h* ≥ 300 mm), in which the FDD occurred in the whole variable domain, except the region with small flange thickness and width. And with the increase in web thickness, the FDD gradually shrank.

To quantitatively describe the FDD under geometry variables of different values, an empirical linear design criterion was proposed herein as Equation (24), where $\left(t_{l},b_{l}\right)$ and $\left(t_{r},b_{r}\right)$ are two points on the linear boundary of FDD.

(24) $$b\geq \frac{b_{l}-b_{r}}{t_{l}-t_{r}}\left(t-t_{r}\right)+b_{r}$$

Define the linear boundary as critical line, and according to Figure 4, the parameters in the critical line can be estimated using Equation (25).

(25) $$\left\{\begin{array}{l} t_{l}=7\\ t_{r}=35\\ b_{l}=\frac{1}{3}\left(h-150\right)+5\left(t_{w}-5\right)+100\\ b_{r}=\left\{\begin{array}{cc} -20\left(t_{w}-5\right)+500 & h\leq 150\\ 100 & h>150 \end{array}\right. \end{array}\right.$$

**Figure 5:**
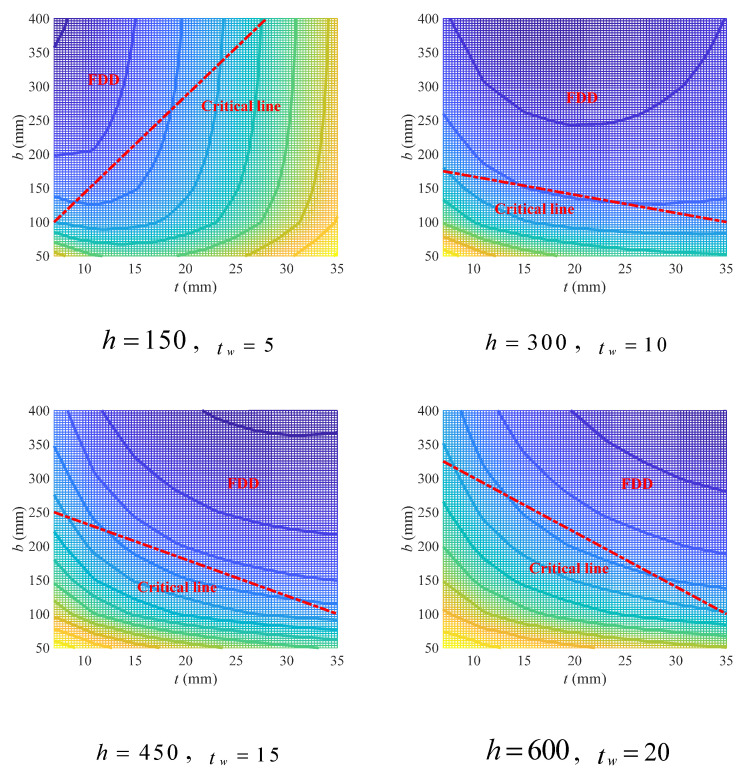
Empirical linear design criterion for flange variables of double-symmetric cross-sections under flexural loads.

Four sets of variables were taken as examples to show the effect of the empirical criterion, as exhibited in Figure 5. In general, the empirical criterion has given a prediction for FDD with acceptable accuracy, even though the prediction tends to be conservative. Using a curve criterion instead of a linear one may be conducive to more precise prediction of FDD, but the linear criterion proposed herein still makes sense as it can be conveniently adopted in practical engineering for fast design with less computation.

**Figure 6:**
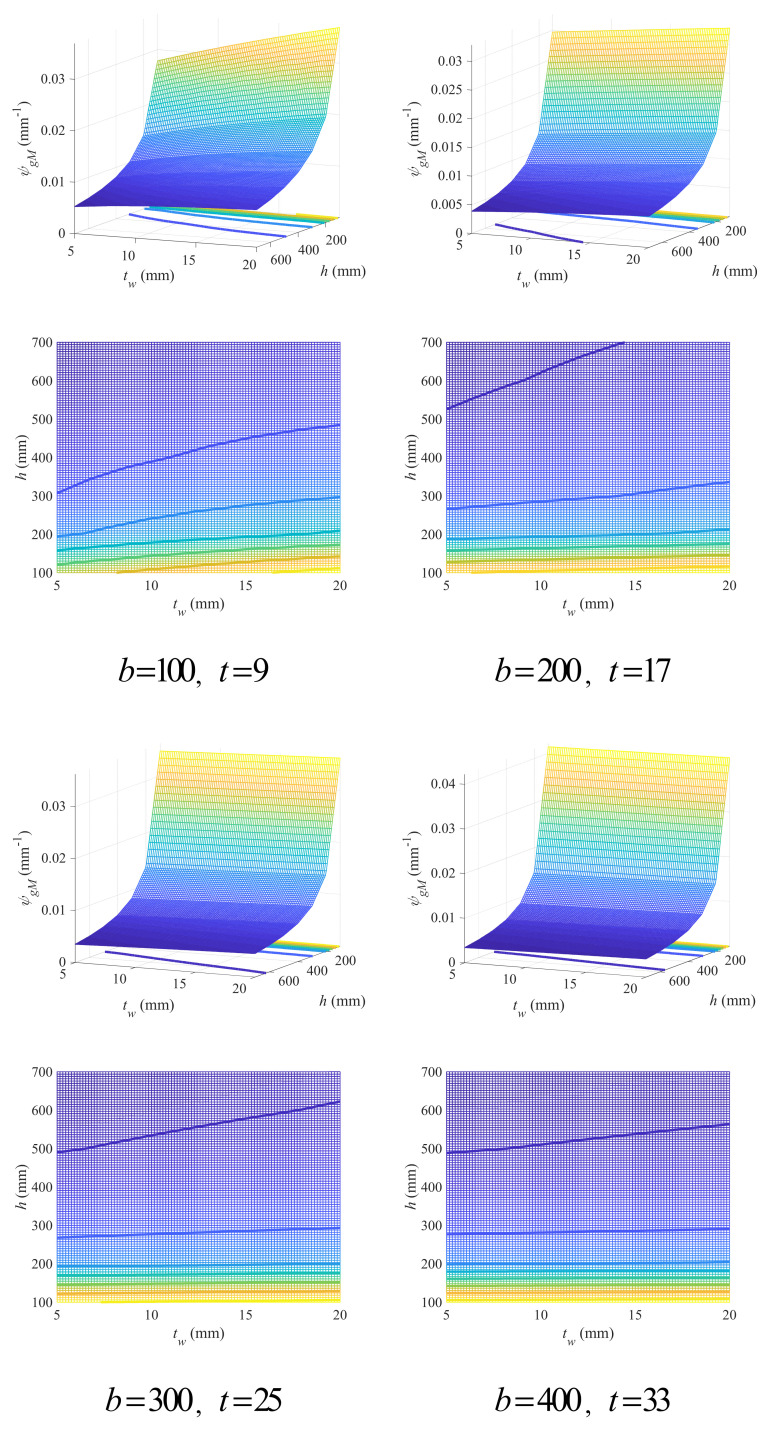
Effects of beam height and web thickness on geometry coefficient under different flange width and thickness (mm).

The variation in the geometry coefficient with beam height and web thickness under different flange variables is shown in Figure 6 (see Figure S2 in Supplementary Material for full figures), which has confirmed some findings above. For instance, the geometry coefficient decreased with the increase in beam height. And as the flange width increased, a slight decrease in the geometry coefficient could also be observed. It was also found that for beams with higher cross-sections, an increase in web thickness will always lead to a larger geometry coefficient, but as the flange thickness increased, the increase in the geometry coefficient with web thickness would get slower, while the flange width seems to have no obvious influence on such variation. For beam cross-sections with lower height, the cases can vary. When flange thickness was small, the geometry coefficient increased with the increase in web thickness. However, such increase was inhibited or even transformed to be decrease as the flange thickness got larger. The flange width also played a similar role, whose increase would lead to slower increase in the geometry coefficient with web thickness.

Contour graphs were also presented in Figure 6 (see Figure S2 in Supplementary Material for full figures). It can be seen that the boundaries of the FDDs are roughly linear, and a small beam height is generally unacceptable, regardless of web thickness. With the increase in flange thickness, the FDDs get enlarged, while the flange width has almost no effects on FDDs. Accordingly, an empirical linear design criterion was also given herein for beam height and web thickness as Equation (26), where $\left(t_{wl},h_{l}\right)$ and $\left(t_{wr},h_{r}\right)$ are two points on the critical line. Based on Figure 4, the parameters in the critical line can be estimated according to Equation (27).

(26) $$h\geq \frac{h_{l}-h_{r}}{t_{wl}-t_{wr}}\left(t_{w}-t_{wr}\right)+h_{r}$$

(27) $$\left\{\begin{array}{l} t_{wl}=5\\ t_{wr}=20\\ h_{l}=200\\ h_{r}=-\frac{25}{8}\left(t-9\right)+300 \end{array}\right.$$

**Figure 7:**
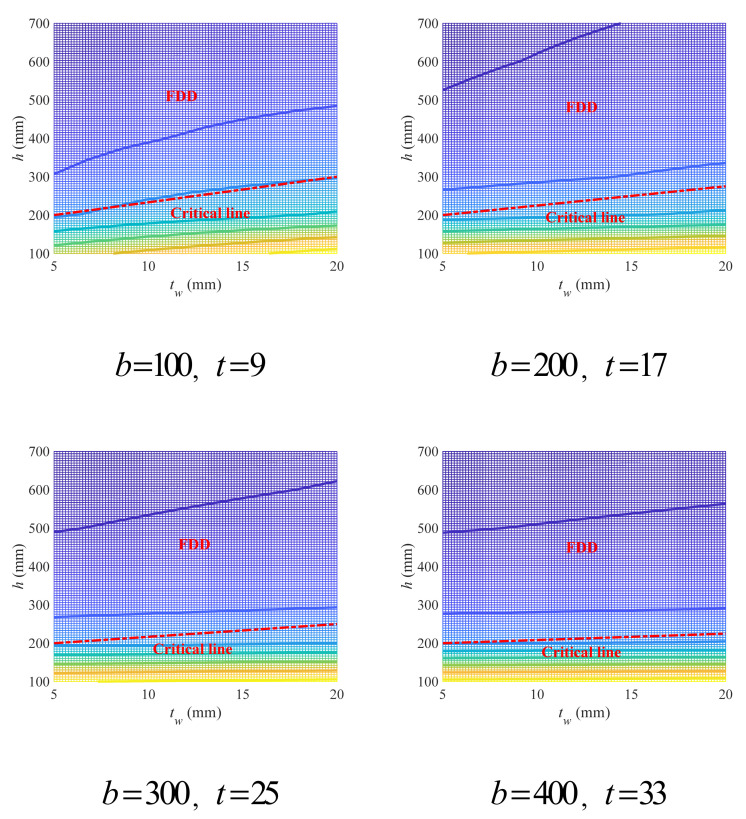
Empirical linear design criterion for beam height and web thickness of double-symmetric cross-sections under flexural loads.

The proposed design criterion was validated on several cases, as shown in Figure 7, which can well describe the FDD. Compared with the criterion for flange variables, the one for beam height and web thickness presented herein has shown much higher accuracy, mainly due to the roughly linear morphology of the contours.

##### Single-Symmetric Cross-Section

As for single-symmetric cross-sections, there are five independent variables, namely the upper flange width ($b_{1}$) and thickness ($t_{1}$), the lower flange width ($b_{2}$) and thickness ($t_{2}$), as well as the beam height (h). Variable $t_{w}$, i.e., the web thickness, can be obtained with the above five variables using Equation (13). Theoretical analysis has revealed that the geometry variables of upper flange ($b_{1}$ and $t_{1}$) and those of lower flange ($b_{2}$ and $t_{2}$) are conjugate variables for the geometry coefficient derived under flexural conditions, meaning that an exchange of their values will not cause any changes in the value of the geometry coefficient. Therefore, the effects of upper flange variables on the geometry coefficient are completely equivalent to those of lower flange variables. It should be noted that different from the four independent variables in double-symmetric cross-sections, the values of the five in single-symmetric cross-sections cannot be freely assigned. Instead, a constraint as Equation (28) must be satisfied to ensure the web thickness falls in the range given in Table 1. And also, there is $t_{1}\neq t_{2}$.

(28) $$4.5\leq \left|\frac{b_{1}t_{1}\left(h-t_{1}\right)-b_{2}t_{2}\left(h-t_{2}\right)}{\left(t_{1}-t_{2}\right)\left(h-t_{1}-t_{2}\right)}\right|\leq 21$$

**Figure 8:**
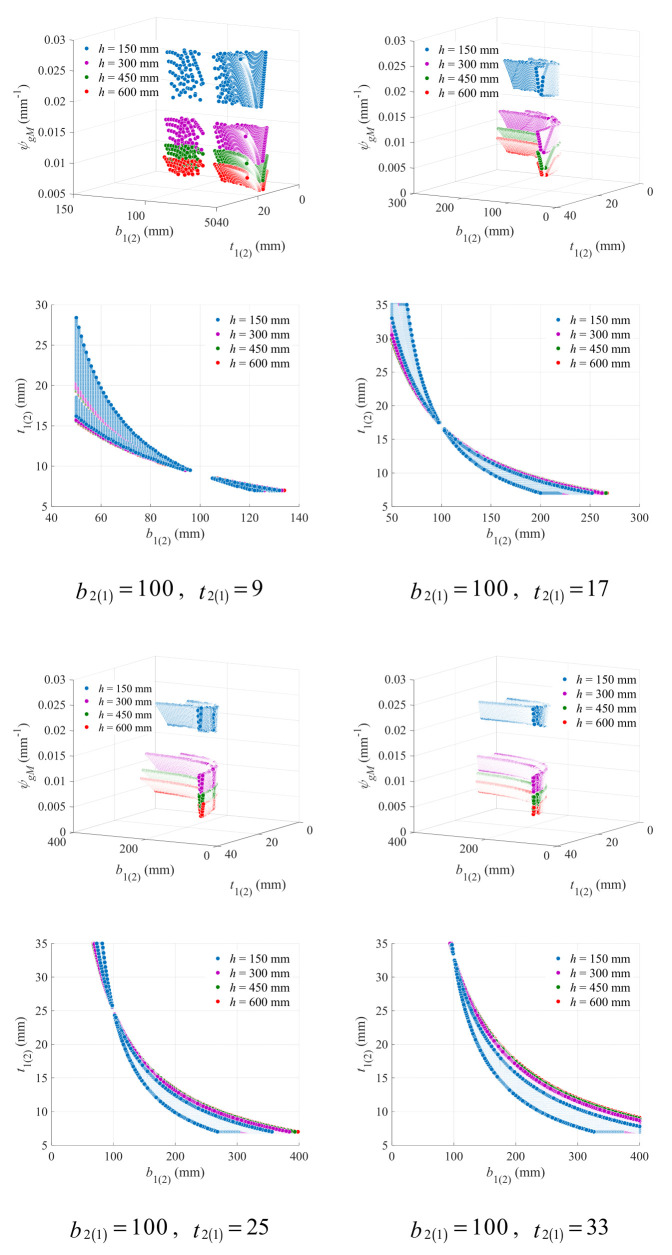
Effects of upper (lower) flange width and thickness on geometry coefficient under different beam height and lower (upper) flange thickness (mm).

The variation in the geometry coefficient with upper (lower) flange width and thickness under different lower (upper) flange thickness and beam height has been plotted in Figure 8, in which the lower (upper) flange width was kept to be 100 mm. It is obvious that the increase in beam height will lead to significant decrease in the geometry coefficient. However, such a decrease in the geometry coefficient is nonuniform, which is much faster when the beam height is small. That has led to an interesting phenomenon: for each given set of lower (upper) flange width and thickness, the four curves corresponding to four different beam heights can be classified into two clusters with the curve for beam height of 150 mm as one cluster hanging over the other three curves as another cluster. The increase in lower (upper) flange thickness will also cause a slight decrease in the geometry coefficient that is not as obvious as beam height.

The three-dimensional curves formed by the coordinate points of $\left(b_{1(2)},t_{1(2)},\varphi_{gM}\right)$ have all shown a “V” form, just like horizontal troughs with V-form cross-sections. For each V-form curve, i.e., a specific beam height, the bottom is roughly on a horizontal plane, and all its V-form cross-sections (defined as curve height), regardless of their positions, are nearly the same. With the increase in lower (upper) flange thickness, the curve height was observed to decrease accordingly.

The plane graphs of the V-form curves have also been shown in Figure 8, and it can be seen that for each curve the widths of its V-form cross-sections are not constant. Generally, the width gradually decreased with the increase in upper (lower) flange thickness and finally reached its minimum (nearly zero) as the upper (lower) flange thickness infinitely approached the lower (upper) flange thickness. Once the upper (lower) flange thickness exceeded the lower (upper) flange thickness and went on to get larger, the curve width would start to increase.

Obviously, the optimal design for upper (lower) flange width and thickness (i.e., the design with the lowest geometry coefficient) falls on the horizontal projections of the bottom of the V-form curves. Define the horizontal projection of the curve bottom as critical line, and the points on or near the critical line are recommended. Such criterion can be expressed as Equation (29), where $c\left(b_{1(2)}\right)$ represents the function of the critical line, and δ is a small positive number.

(29) $$\left|t_{1(2)}-c\left(b_{1(2)}\right)\right|\leq \delta$$

Considering that a linear model can have significant error in description, a hyperbolic model was established instead for the critical line, as shown in Equation (30).

(30) $$c\left(b_{1(2)}\right)=\frac{a}{b_{1(2)}+d}$$

**Figure 9:**
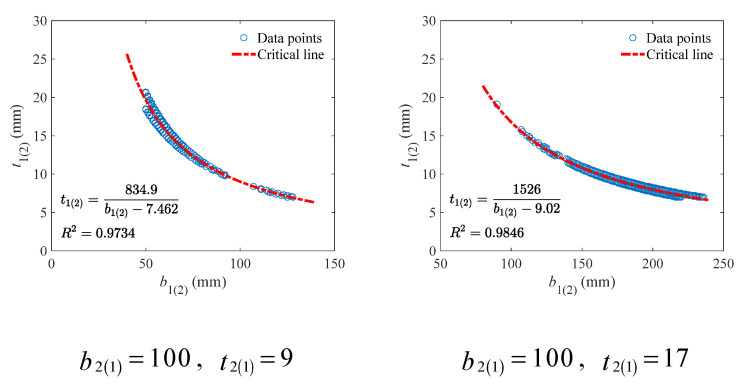

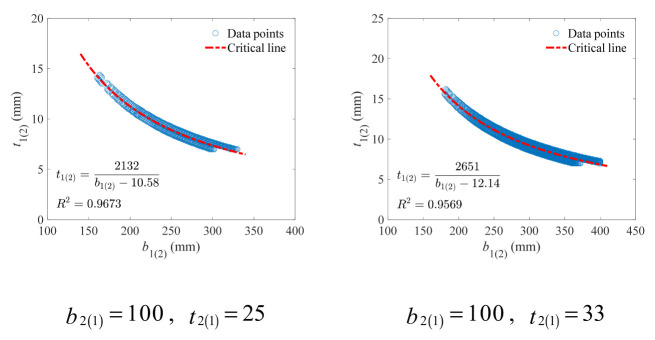
Fitting results of the hyperbolic critical line for the upper (lower) flange width and thickness under different lower (upper) flange thickness (mm).

Since according to Figure 8 the critical lines under different beam height are nearly the same, the data points from the bottom of the V-form curve under the beam height of 150 mm were taken for validation of the proposed hyperbolic model, as shown in Figure 9. It can be seen that the hyperbolic model can well describe the critical lines, with the *R*^2^ values always higher than 0.95.

**Figure 10:**
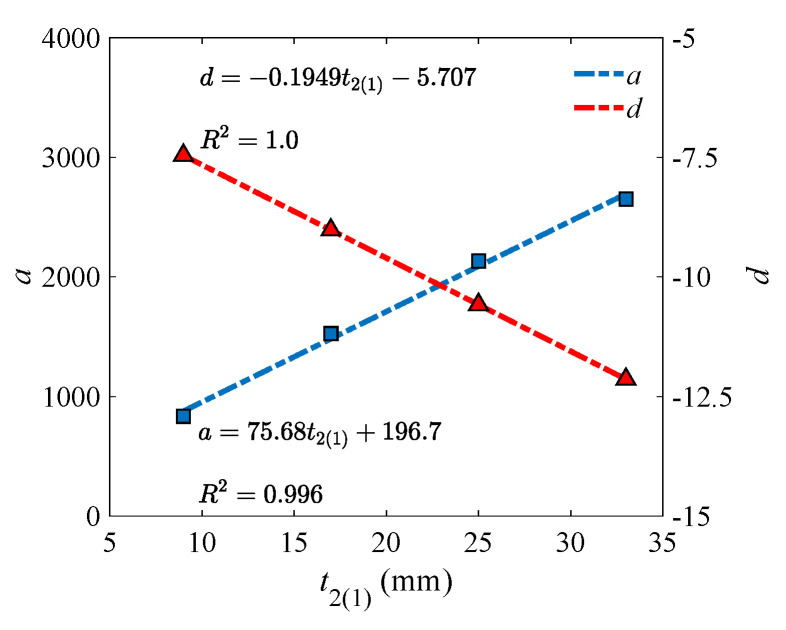
Correlation between model parameters and lower (upper) flange thickness.

To find the model parameters (i.e., a and d in Equation (30)) under different lower (upper) flange thickness, their relation was investigated. As shown in Figure 10, a strong correlation could be observed. That means the model parameters can be predicted accurately through Equation (31).

(31) $$\left\{\begin{array}{l} a=75.68t_{2(1)}+196.7\\ d=-0.1949t_{2(1)}-5.707 \end{array}\right.$$

**Figure 11:**
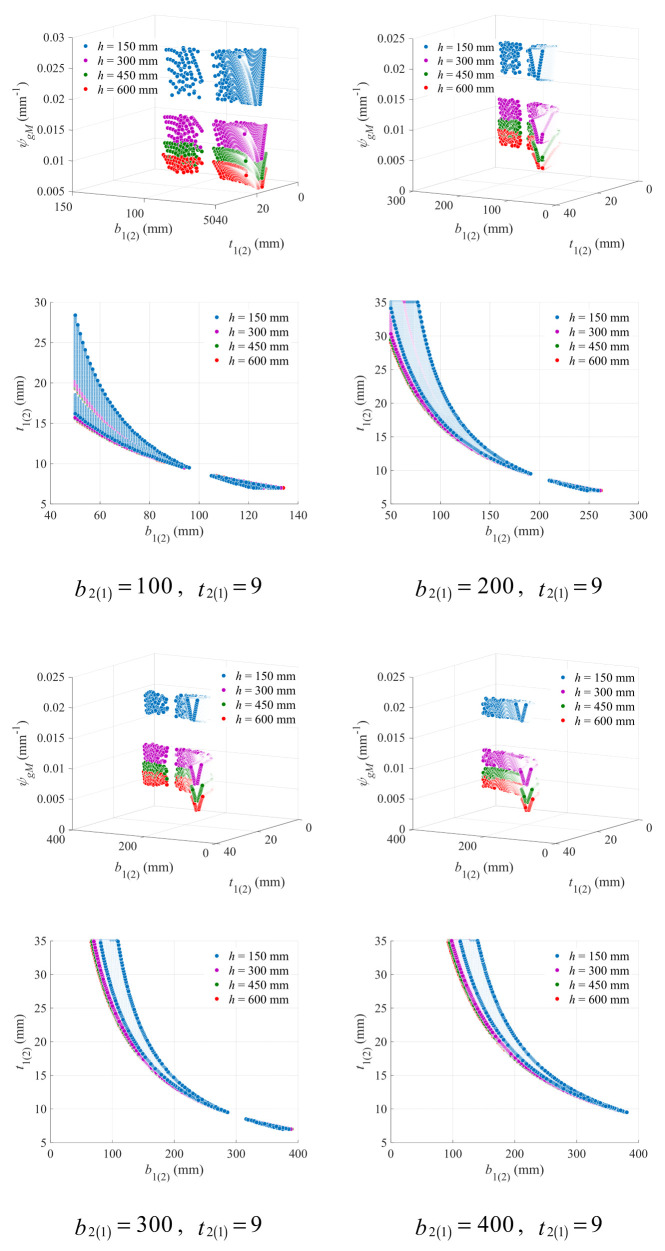
Effects of upper (lower) flange width and thickness on geometry coefficient under different beam height and lower (upper) flange width (mm).

The variation in the geometry coefficient with upper (lower) flange width and thickness under different lower (upper) flange width and beam height was plotted in Figure 11, with the lower (upper) flange thickness kept to be 9 mm. Effects of the lower (upper) flange width are roughly the same as those of the lower (upper) flange thickness, and all phenomena previously found in Figure 8 can also be observed in Figure 11.

**Figure 12:**
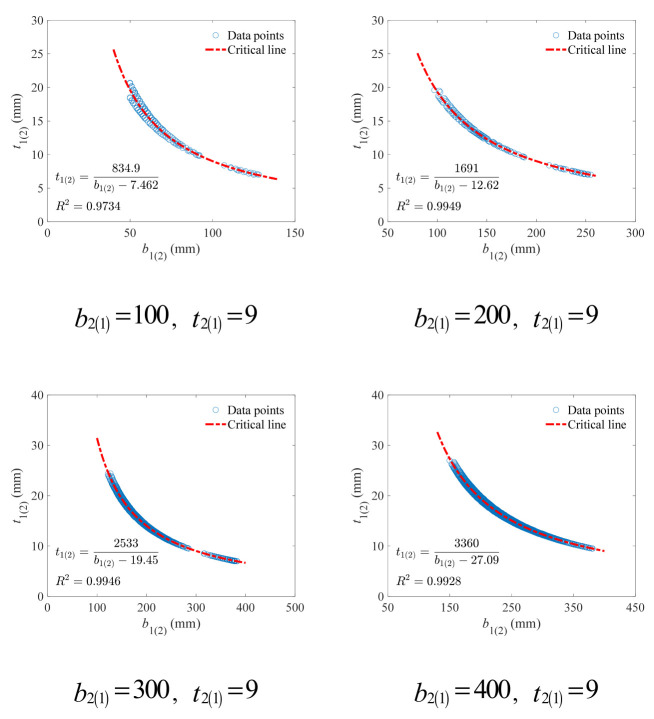
Fitting results of the hyperbolic critical line for the upper (lower) flange width and thickness under different lower (upper) flange width (mm).

Similarly, the critical line was described with the hyperbolic model as Equation (30), and the fitting results on the data points from the bottom of the V-form curve under the beam height of 150 mm were presented in Figure 12. The hyperbolic model still shows extremely high accuracy (*R*^2^ > 0.97), indicating its stable effectiveness under different values of flange variables.

**Figure 13:**
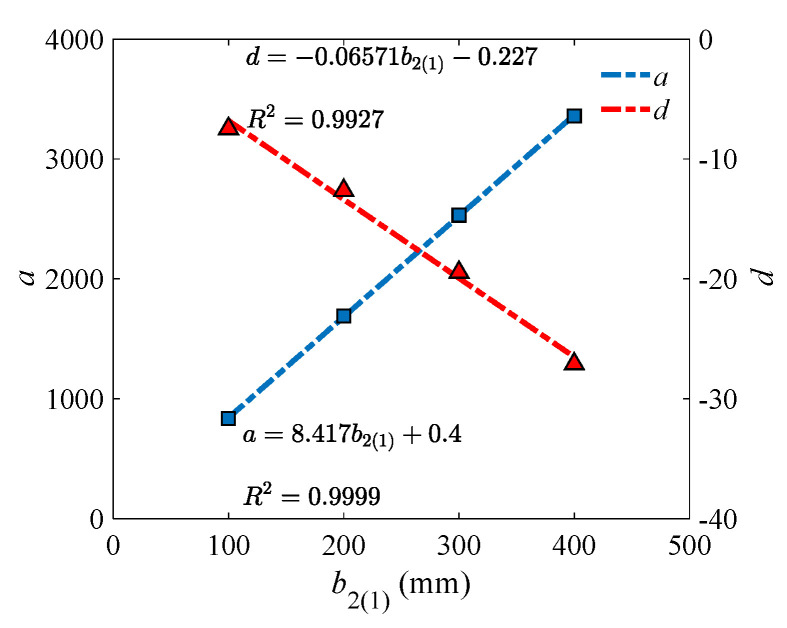
Correlation between model parameters and lower (upper) flange width.

A strong correlation can still be observed between model parameters and lower (upper) flange width, as exhibited in Figure 13. With Equation (32), the parameters can be precisely obtained.

(32) $$\left\{\begin{array}{l} a=8.417b_{2(1)}+0.4\\ d=-0.06571b_{2(1)}-0.227 \end{array}\right.$$

#### 3.1.2. Criteria Governed by Ultimate Shear Strength

**Figure 14:**
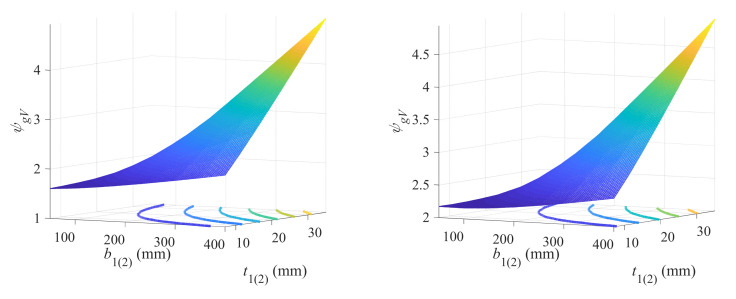

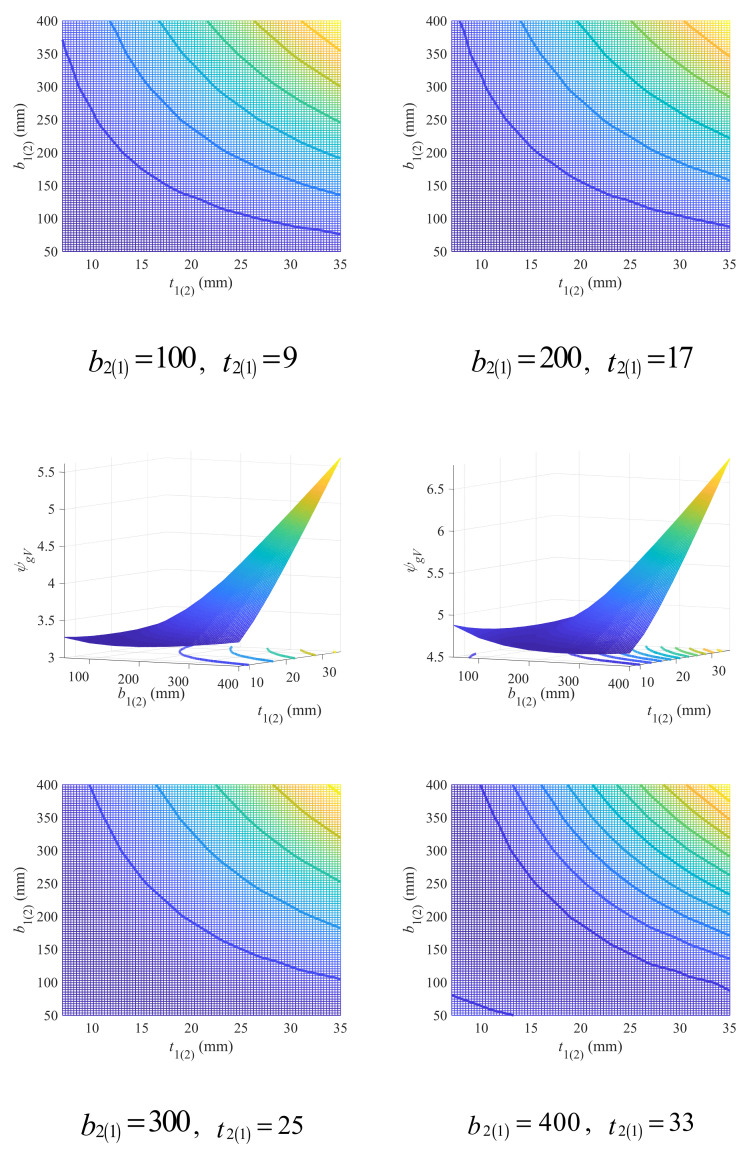
Effects of upper (lower) flange width and thickness on geometry coefficient under different lower (upper) flange width and thickness (mm).

The ultimate shear strength of I/H-beam is not affected by the symmetry of cross-section, and thereby governed by six independent variables including the upper flange width ($b_{1}$) and thickness ($t_{1}$), the lower flange width ($b_{2}$) and thickness ($t_{2}$), the beam height (h), as well as the web thickness ($t_{w}$). Similar to the case for single-symmetric cross-section, the upper flange variables ($b_{1}$ and $t_{1}$) and the lower flange variables ($b_{2}$ and $t_{2}$) are still conjugates for geometry coefficient derived in shear conditions, and therefore the variation in the geometry coefficient was plotted as Figure 14 (see Figure S3 in Supplementary Material for full figures) to show the effects of upper (lower) flange width and thickness under different lower (upper) flange width and thickness, where the beam height was fixed to be 400 mm and the web thickness to be 13 mm. It can be observed that the increase in either upper or lower flange thickness would lead to an increase in the geometry coefficient, and so would the upper and lower flange width. In this case, increasing the width or thickness of either upper or lower flange is not a good choice for beams mainly bearing shear loads.

As clearly shown in the contour graphs of Figure 14 (see Figure S3 in Supplementary Material for full figures), the FDD falls in the region with either small upper (lower) flange width or thickness. And as the lower (upper) flange thickness increased, the FDD gradually got larger. While the lower (upper) flange width was found to have no obvious impact on FDD. To describe the FDD under different flange variables, an empirical linear design criterion as Equation (33) was proposed based on Figure 14, of which the parameters were suggested as Equation (34). As before, $\left(t_{1\left(2\right)l},b_{1\left(2\right)l}\right)$ and $\left(t_{1\left(2\right)r},b_{1\left(2\right)r}\right)$ are two points on the critical line.

(33) $$b_{1\left(2\right)}\leq \frac{b_{1\left(2\right)l}-b_{1\left(2\right)r}}{t_{1\left(2\right)l}-t_{1\left(2\right)r}}\left(t_{1\left(2\right)}-t_{1\left(2\right)r}\right)+b_{1\left(2\right)r}$$

(34) $$\left\{\begin{array}{l} t_{1\left(2\right)l}=15\\ t_{1\left(2\right)r}=35\\ b_{1\left(2\right)l}=400\\ b_{1\left(2\right)r}=\frac{5}{2}\left(t_{2\left(1\right)}-9\right)+140 \end{array}\right.$$

**Figure 15:**
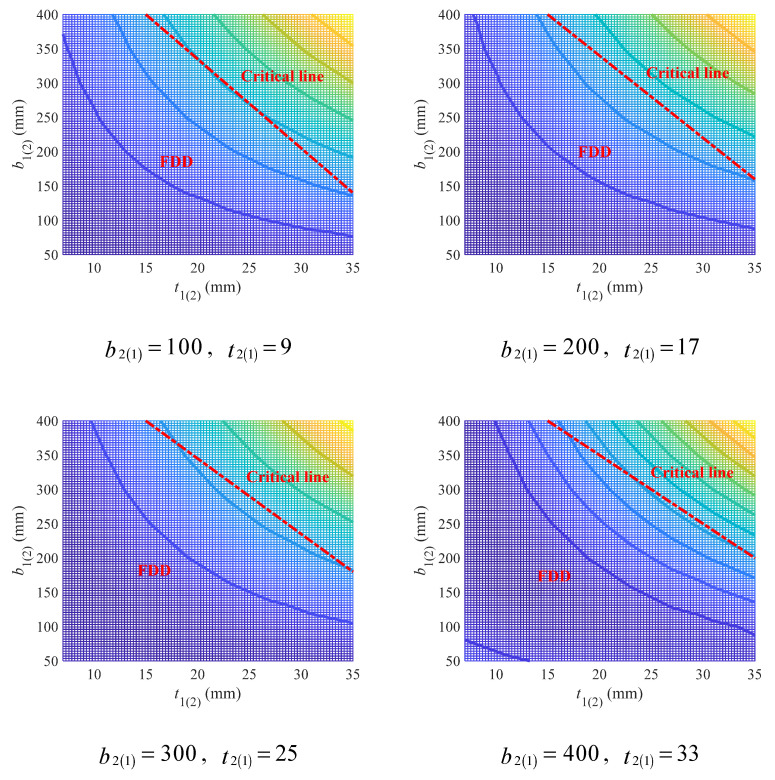
Empirical linear design criterion for upper (lower) flange width and thickness under shear loads.

The FDD delineated by the above critical line was exhibited in Figure 15, which is quite acceptable. The proposed linear criterion is effective, even though the contours are non-linear.

**Figure 16:**
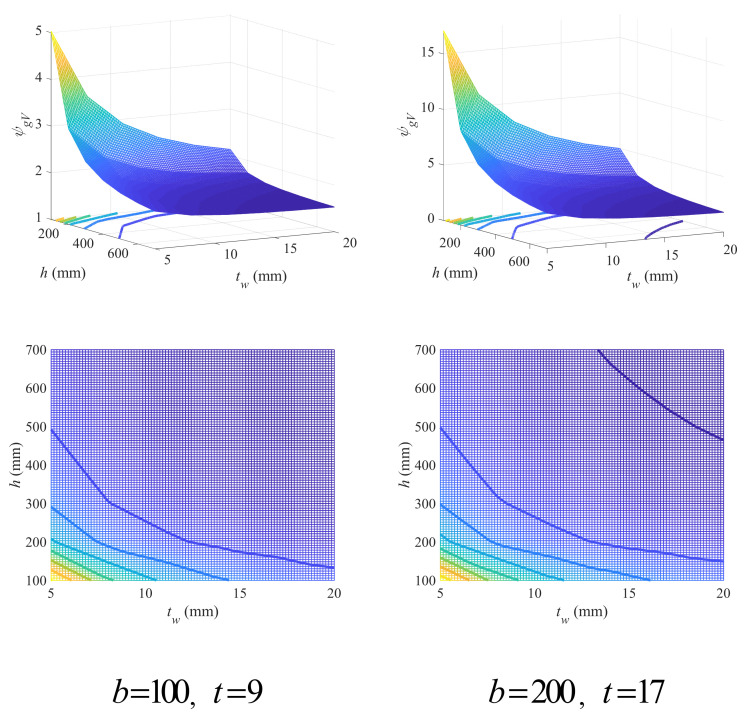

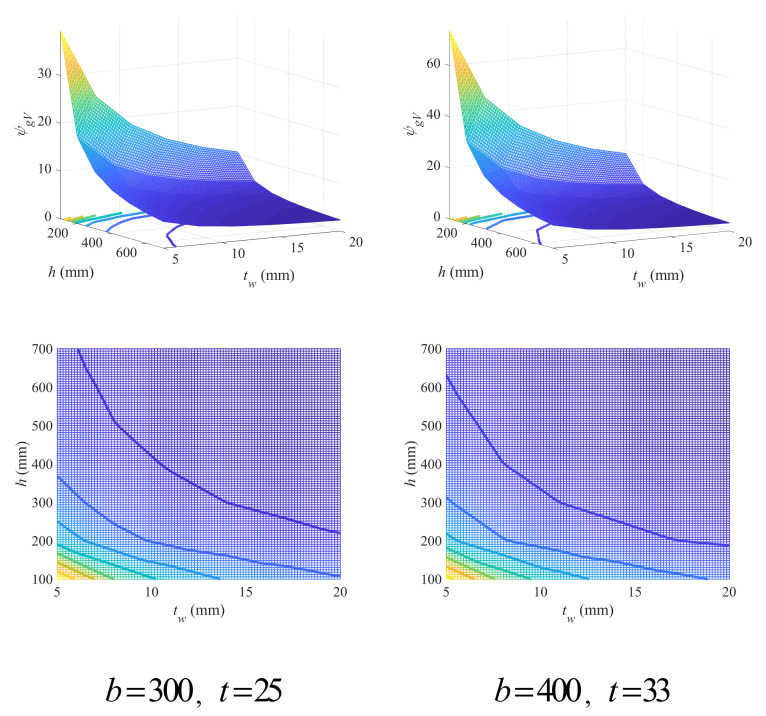
Effects of beam height and web thickness on geometry coefficient under different flange width and thickness (mm).

The variation in the geometry coefficient with beam height and web thickness under different flange width and thickness is shown in Figure 16 (see Figure S4 in Supplementary Material for full figures). For convenience, the width as well as the thickness of the upper and lower flange were kept the same. It is easy to see that the increase in either beam height or web thickness will bring a lower geometry coefficient. While for flange width and thickness, just as found in Figure 14, their increase will cause a higher geometry coefficient. Therefore, beams mainly bearing shear loads are suggested with larger beam height or web thickness and in the meanwhile smaller flange width and thickness.

The contour graphs in Figure 16 show that the FDD falls in the region with either large beam height or web thickness (see Figure S4 in Supplementary Material for full figures). However, the FDD is not sensitive to the variation in flange variables and varies little under different flange width and thickness. In this case, an empirical design criterion was proposed to be fixed as Equation (35).

(35) $$h\geq -20t_{w}+400$$

**Figure 17:**
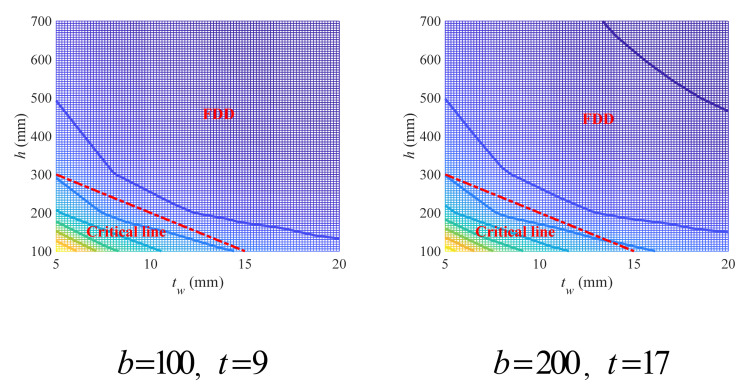

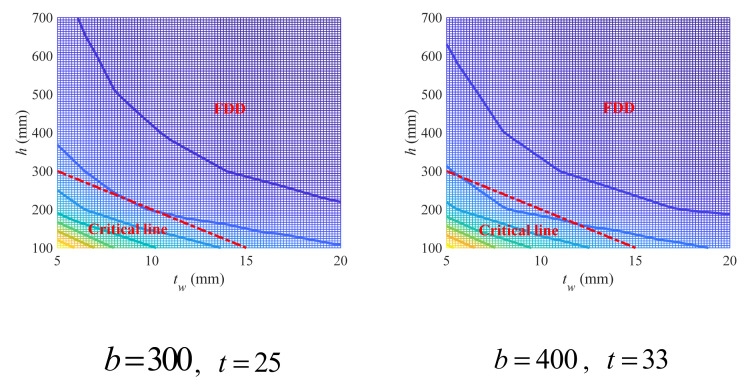
Empirical linear design criterion for beam height and web thickness under shear loads.

The criterion given by Equation (35) was shown in Figure 17, which has presented a good description for FDD in all examples.

### 3.2. Material Criteria

As discussed in Section 2.3, the material coefficient ($\gamma_{m}$) can always be expressed in the form of Equation (36), where $C_{s}$ is the carbon emissions generated per unit volume of steel, and $f_{s}$ represents a specific type of strength depending on load conditions, such as the tensile strength in Equation (22) or shear strength in Equation (23). Since lower CCR means a more sustainable design, the material coefficient is also expected to be smaller. Therefore, steel with higher strength ($f_{s}$) is suggested for beam fabrication. In fact, such inference has confirmed some previous findings [51]. It should be noted that pursuing higher strength in steel selection is only a generalized recommendation as the strength of steel is measured by many indicators. A material cannot always achieve superior performance simultaneously in terms of all strength indicators, and which indicator to focus on should be determined based on the actual load conditions. For instance, if a beam mainly bears flexural loads, then tensile strength should be emphasized and the steel with higher tensile strength should be adopted. Instead, if a beam is faced with a complex load condition, then a comprehensive consideration for multiple strength indicators will be necessary.

(36) $$\gamma_{m}=\frac{C_{s}}{f_{s}}$$

Another recommendation is to reduce the carbon emissions ($C_{s}$) in the production of steel beams, including the carbon emissions in the upstream production of raw steel as well as those in the fabrication process [67]. Compared with the fabrication process, the upstream production of raw steel is a main contributor [68]. Generally, two stages are involved in the production of raw steel, that is, the selection of raw materials and the smelting of steel [69]. As for the raw materials, using scrap steel in place of pig iron was found to be able to significantly reduce the carbon emissions [70,71]. While for the smelting of steel, promoting energy-saving technologies is believed to be the most effective measure [72], and using large-scale equipment, hydrogen reduction ironmaking technology, as well as an electric arc furnace is recommended [73]. As a summary, steel produced from recycled scrap using energy-saving technologies is suggested for beam fabrication. When it comes to the following beam fabrication process, where the carbon emissions mainly come from the consumption of energy and material [67], new prototyping techniques like cold forging [74], laser remelting based additive manufacturing [75], as well as the wire arc additive manufacturing in conjunction with topology optimization [76] are suggested in substitution for conventional ones so that the energy and material loss can be lowered.

Strictly speaking, the geometry coefficient and material coefficient are not absolutely independent with each other, even though the former has nothing to do with the type of material used. As mentioned above, the carbon emissions involved in beam fabrication process, which is a constituent part of $C_{s}$ in material coefficient, mainly come from the energy and material consumption. Since the changes in the geometry coefficient will bring different beam cross-sections, inevitably leading to different energy and material consumption in fabrication, it will definitely cause an impact on material coefficient. However, such impact can be slight and is acceptable to neglect in real design.

## 4. Conclusions

In this study, a comprehensive index named carbon-capacity ratio was developed, based on which the geometry coefficient and material coefficient were derived. Quantitative and qualitative investigation were respectively conducted on the geometry coefficient and material coefficient, and the geometry as well as material criteria for low-carbon I/H-beam design were proposed. Conclusions were summarized as follows:

(1) For double-symmetric cross-sections bearing flexural loads, larger flange width and beam height are recommended, while the thickness of flange and web can form a coupling effect with other geometry variables and should be determined accordingly. The FDD for flange width and thickness depends heavily on beam height, which, together with that for beam height and web thickness, can be predicted through empirical linear design criteria with acceptable accuracy.
(2) For single-symmetric cross-sections bearing flexural loads, increasing beam height as well as flange width and thickness are all conducive to the low-carbon design, but using large beam height is the most effective. The FDD for upper (lower) flange width and thickness falls on the neighborhood of the critical line, which is mainly affected by lower (upper) flange width and thickness, while the influence of beam height is not that significant. The critical line can be well described using a hyperbolic model, with all model parameters showing strong linear correlations with both lower (upper) flange width and thickness.
(3) For cross-sections bearing shear loads, increasing beam height and web thickness is beneficial, while increasing the width or thickness of either upper or lower flange is not a good choice. The FDD for upper (lower) flange width and thickness is affected by the thickness of lower (upper) flange, but the width of lower (upper) flange was found to have no obvious impact. The FDD for beam height and web thickness is not sensitive to the variation in flange variables. Both the FDD for upper (lower) flange width and thickness and the one for beam height and web thickness can be predicted through empirical linear design criteria with relatively high accuracy.
(4) For materials used in beams, steel with higher strength or that produced from recycled scrap using energy-saving technologies is suggested. Meanwhile, new prototyping techniques with lower energy and material loss are recommended for the beam fabrication process.

The FDD for I/H-beams under different load conditions has been summarized in Table 2, which can serve as the constraints in the low-carbon design of steel I/H-beams. These constraints have defined the relationship between geometry variables that must be satisfied and have nothing to do with specific design requirements like the desired bearing capacity or the limits on carbon emissions. Since the FDD is obtained based on the fundamental mechanics of materials, it is broad-spectrum and compatible with all current codes or standards for steel structures, that is, it can be adopted as supplementary provisions in the structural codes of different countries that further restrict the design space to add environmental considerations. That makes sense as promoting sustainable design through codes or regulations is likely to have a more immediate effect [77], particularly when implemented in larger scale. For I/H-beams with more complex load conditions, for instance, those bearing both shear and flexural loads, the FDD derived under purely shear and purely flexural conditions can be applied together in the low-carbon design, as the loads can be decomposed into shear loads and flexural loads. In addition to the FDD, this study also exhibits good generalization in terms of methodology: the proposed CCR as well as the way the geometry and material coefficients were derived and evaluated are applicable to the components in diverse geometry or those made of other materials like concrete [78] or various mortars [79,80,81,82,83,84], and the criteria for other goal-oriented design can be established in a similar way. In other words, this study has presented a systematic method for handling materials considering different performance indices. Taking the low-pollution design of steel I/H-beams as an example, the only difference is to change the variable $C_{s}$, i.e., the carbon emissions generated per unit volume of steel, in the material coefficient for the amount of pollutants generated (e.g., NO*_x_*) per unit volume of steel, while the geometry coefficient remains unchanged. In this case, the geometry criteria derived in this study are still effective, while the material criteria simply change to be using low-pollution raw materials and fabrication techniques to produce steel with higher strength. Certain specific recommendations can be given according to the analysis on the material coefficient, such as coating the component with a photocatalyst capable of degrading the pollutants [85].

Despite the contributions, this study also faces some limitations. In the analysis, the carbon emissions of I/H-beams were assumed to be fully independent of their geometry. However, different geometry may require different manufacturing techniques that consume varying amounts of material and energy to achieve, thus leading to slightly different carbon emissions. Additionally, this study is developed on the fundamental mechanics of materials with simple elastic assumptions. Although that has endowed this study with generality and made the findings compatible with all current structural codes, it brings relatively wider design space as well. That is, using the proposed FDD merely as design constraints is not enough. In addition, some load conditions like twist, though normally less common in structures, are not explored in this study. Future research may expand this study to more diverse load conditions as well as those closer to real engineering. Optimization cases on steel I/H-beams reported in the literature will be reexamined using the criteria proposed in this study to evaluate its significance to current structural codes or standards around the world.

## Figures and Tables

**Table 1:** Ranges of different design variables (mm).

Design Variables	Ranges
Flange width	50~408
Flange thickness	7~35
Beam height	100~700
Web thickness	4.5~21

**Table 2:** Feasible design domain for I/H-beams bearing different loads.

Load	Feasible Design Domain
Flexural	Double-symmetric cross-section: $b\geq \frac{b_{l}-b_{r}}{t_{l}-t_{r}}\left(t-t_{r}\right)+b_{r}$ $h\geq \frac{h_{l}-h_{r}}{t_{wl}-t_{wr}}\left(t_{w}-t_{wr}\right)+h_{r}$ Single-symmetric cross-section: $\left|t_{1(2)}-c\left(b_{1(2)}\right)\right|\leq \delta$ $c\left(b_{1(2)}\right)=\frac{a}{b_{1(2)}+d}$
Shear	$b_{1\left(2\right)}\leq \frac{b_{1\left(2\right)l}-b_{1\left(2\right)r}}{t_{1\left(2\right)l}-t_{1\left(2\right)r}}\left(t_{1\left(2\right)}-t_{1\left(2\right)r}\right)+b_{1\left(2\right)r}$ $h\geq \frac{h_{l}-h_{r}}{t_{wl}-t_{wr}}\left(t_{w}-t_{wr}\right)+h_{r}$

## Data Availability

The original contributions presented in this study are included in the article and Supplementary Materials. Further inquiries can be directed to the corresponding author.

## Supplementary Materials

The following supporting information can be downloaded at: https://www.mdpi.com/article/10.3390/ma18214930/s1, Figure S1: Effects of flange width and thickness on geometry coefficient under different web thickness and beam height (mm); Figure S2: Effects of beam height and web thickness on geometry coefficient under different flange width and thickness (mm); Figure S3: Effects of upper (lower) flange width and thickness on geometry coefficient under different lower (upper) flange width and thickness (mm); Figure S4: Effects of beam height and web thickness on geometry coefficient under different flange width and thickness (mm).
