# Supplementary material for: Geometry and Material Criteria for Low-Carbon Design of I/H-Beams in Sustainable Steel Structures Considering Both Mechanical Properties and Carbon Emissions
Source: Materials (Basel). 2025 Oct 28;18(21):4930. doi: 10.3390/ma18214930 (PMC12609269; doi:10.3390/ma18214930)
Supplement: Supplementary file 1 [file materials-18-04930-s001.zip › materials-3767432-supplementary.pdf]

Supplementary Materials

Geometry and Material Criteria for Low-Carbon Design of I/H-Beams in Sustainable Steel Structures Considering Both Mechanical Properties and Carbon Emissions

Jitao Bai [1,2], Keyong Yang [3], Zhonghao Chen [3], Jiahe Liang [4], Simiao Zhang [5] and Yu Diao [2,6,7,*]

[1] Department of Civil Engineering, Tianjin University, Tianjin 300072, China; jitaobai_123@tju.edu.cn
[2] Key Laboratory of Coast Civil Structures and Safety of Ministry of Education, Tianjin University, Tianjin 300072, China
[3] College of Intelligence and Computing, Tianjin University, Tianjin 300072, China; ykyong@tju.edu.cn (K.Y.); diogeneschen@tju.edu.cn (Z.C.)
[4] Faculty of Architecture, KU Leuven, 9000 Gent, Belgium; liangjiahe117@gmail.com
[5] Software Engineering Institute, East China Normal University, Shanghai 200062, China; smzhang@stu.ecnu.edu.cn
[6] National Science Center for Earthquake Engineering, Tianjin University, Tianjin 300350, China
[7] State Key Laboratory of Hydraulic Engineering Intelligent Construction and Operation, Tianjin University, Tianjin 300350, China
* Correspondence: yudiao@tju.edu.cn

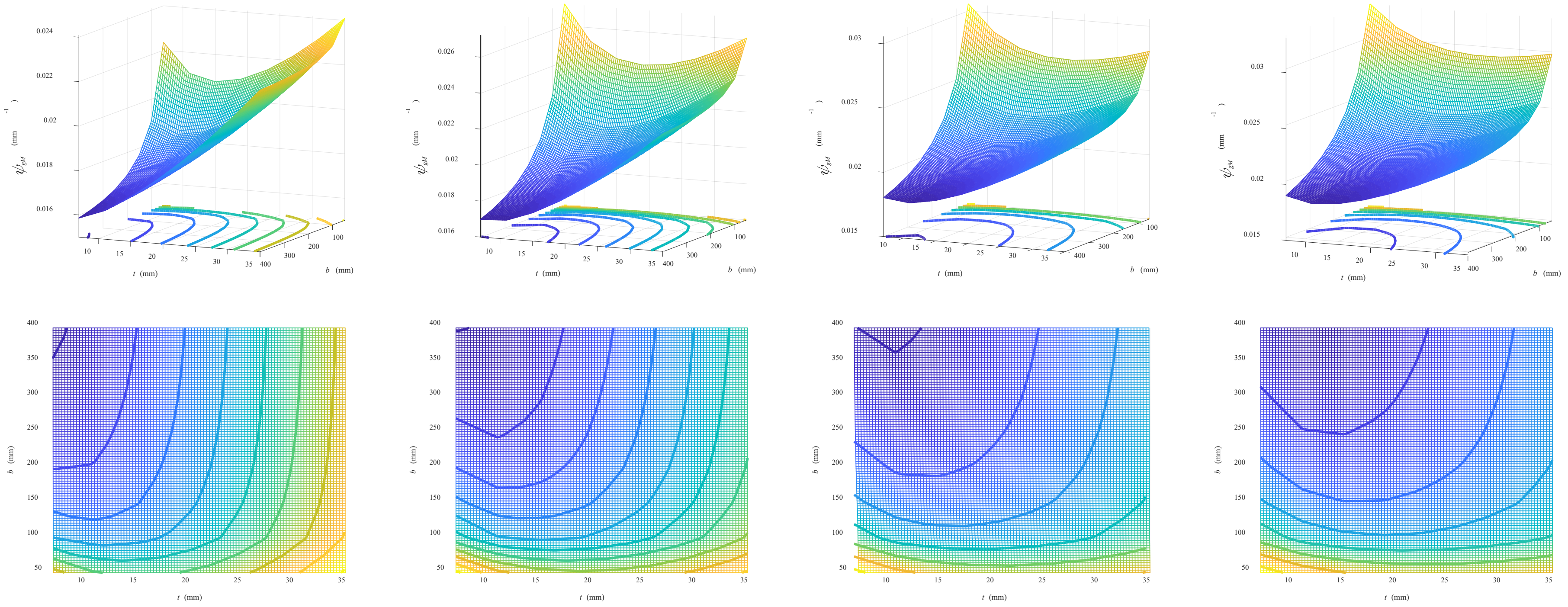

$h = 150\ ,\quad t_w = 5$ $h = 150\ ,\quad t_w = 10$ $h = 150\ ,\quad t_w = 15$ $h = 150\ ,\quad t_w = 20$

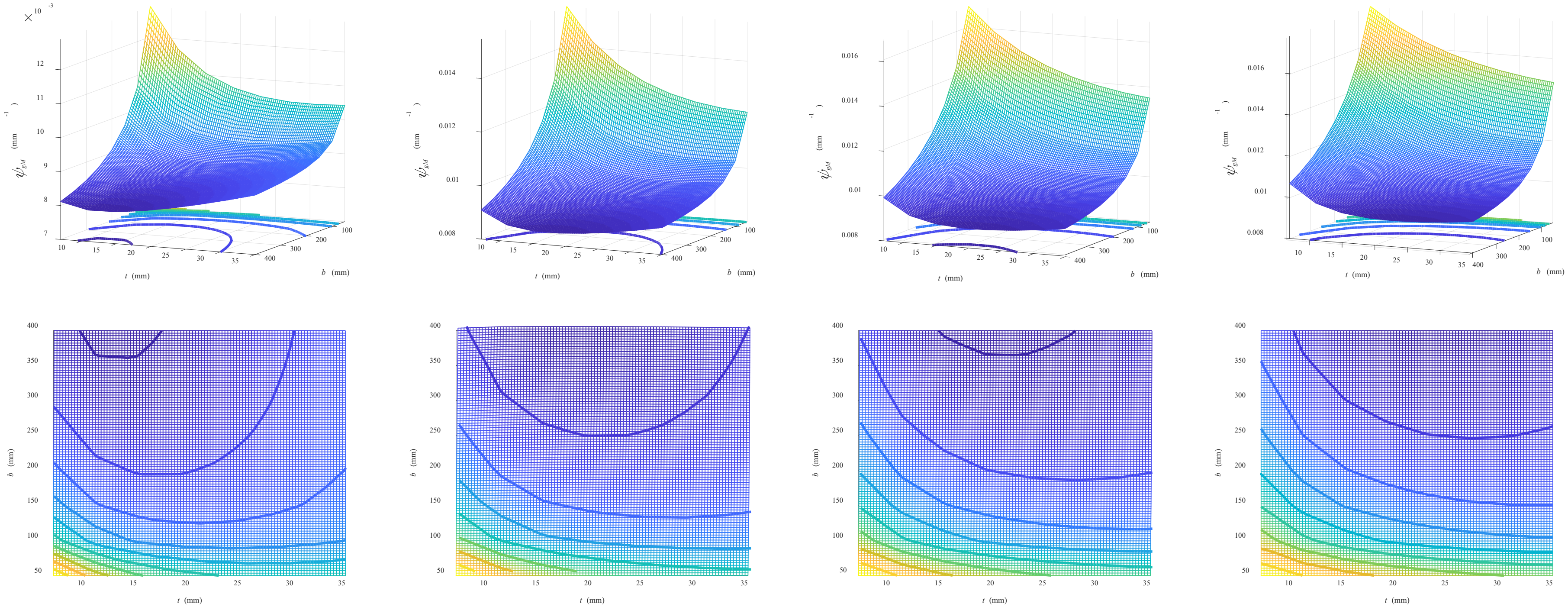

$h = 300$, $t_w = 5$ $h = 300$, $t_w = 10$ $h = 300$, $t_w = 15$ $h = 300$, $t_w = 20$

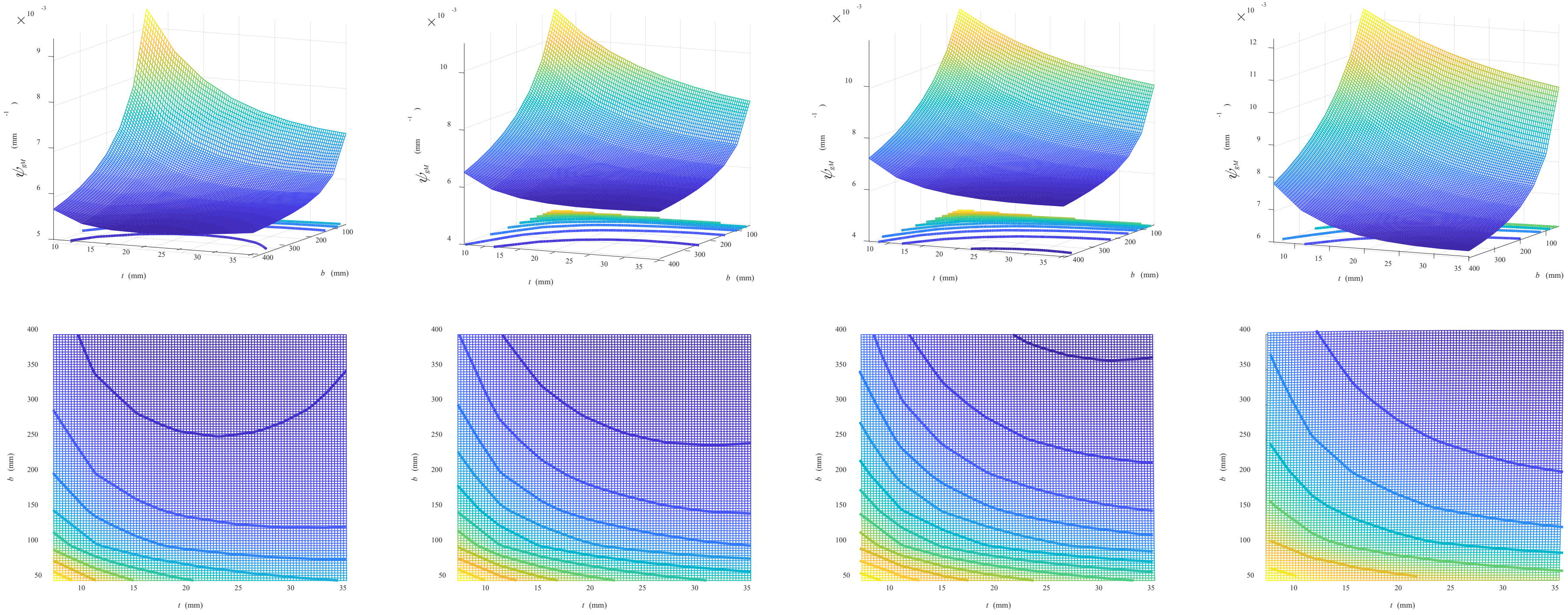

$h = 450$, $t_w = 5$ $h = 450$, $t_w = 10$ $h = 450$, $t_w = 15$ $h = 450$, $t_w = 20$

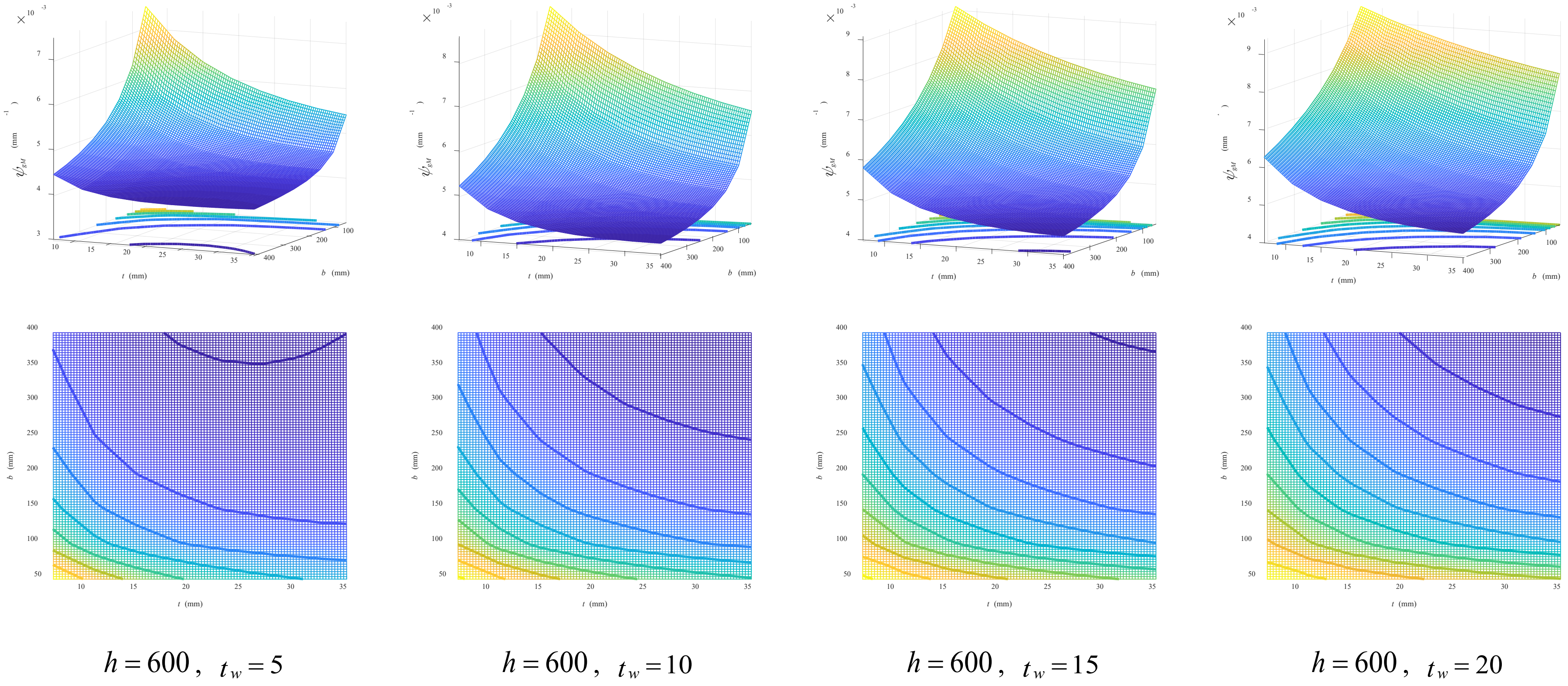

Figure S1. Effects of flange width and thickness on geometry coefficient under different web thickness and beam height (mm)

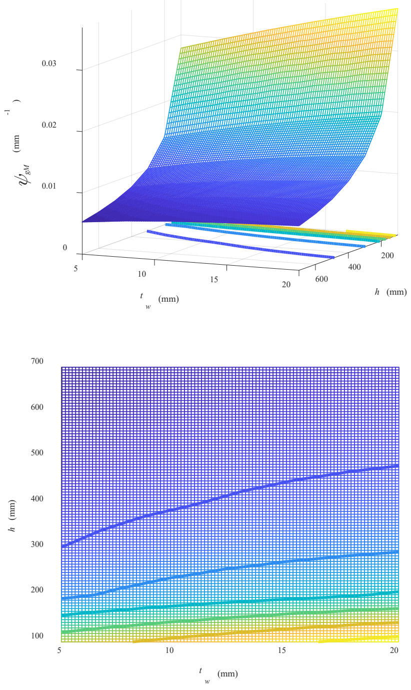

$b = 100$, $t = 9$

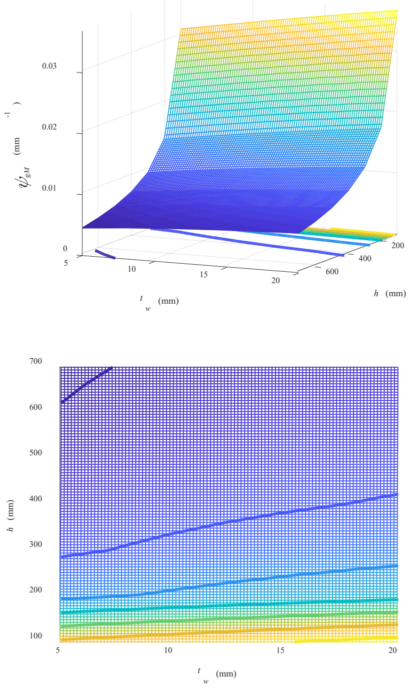

$b = 100$, $t = 17$

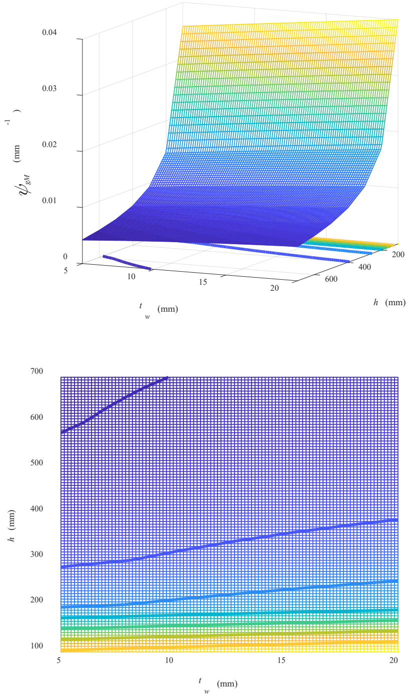

$b = 100$, $t = 25$

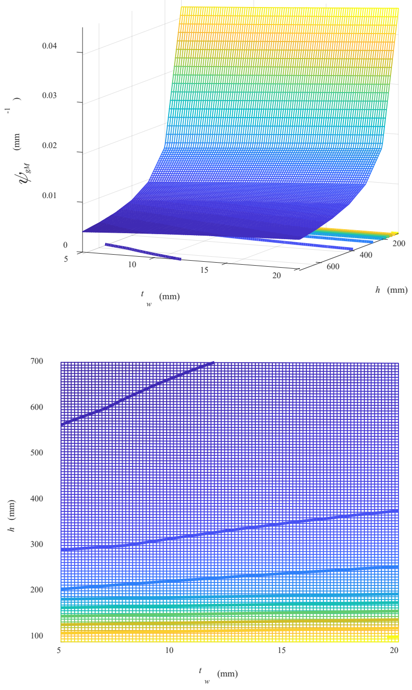

$b = 100$, $t = 33$

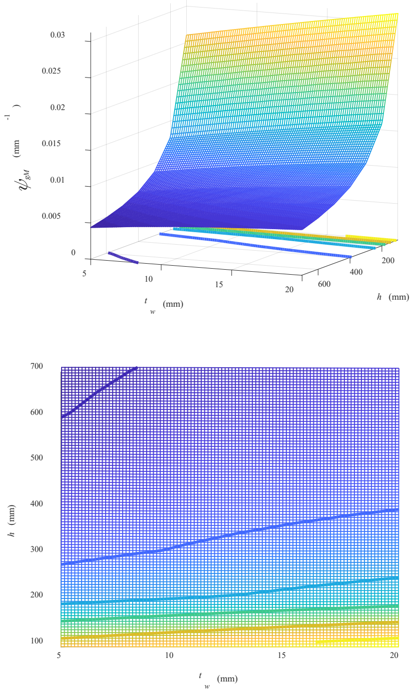

$b = 200$, $t = 9$

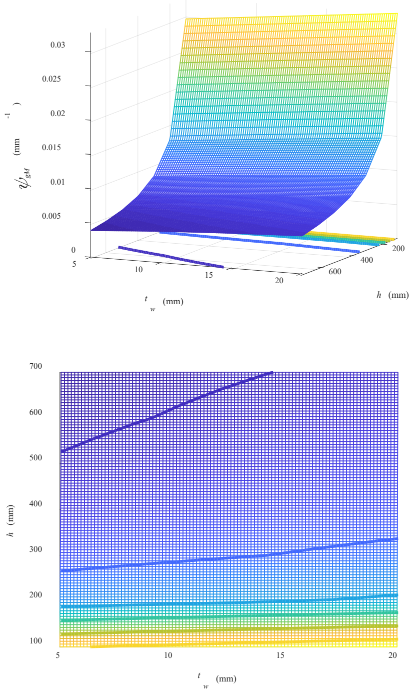

$b = 200$, $t = 17$

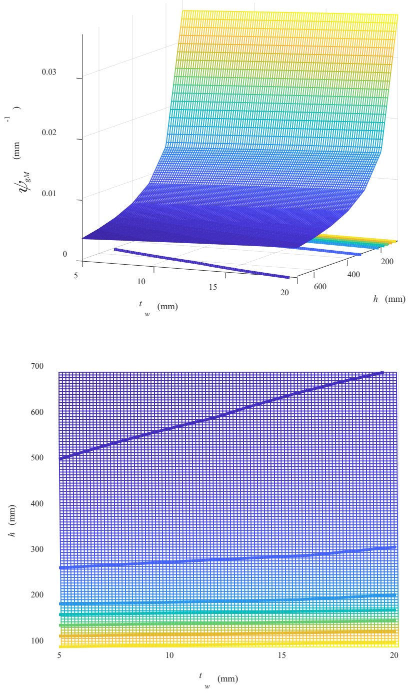

$b = 200$, $t = 25$

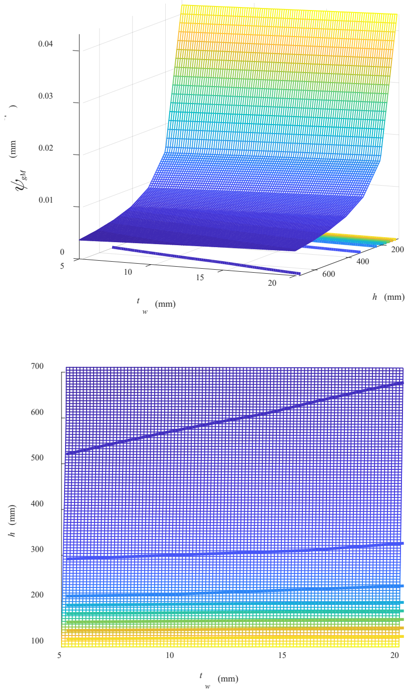

$b = 200$, $t = 33$

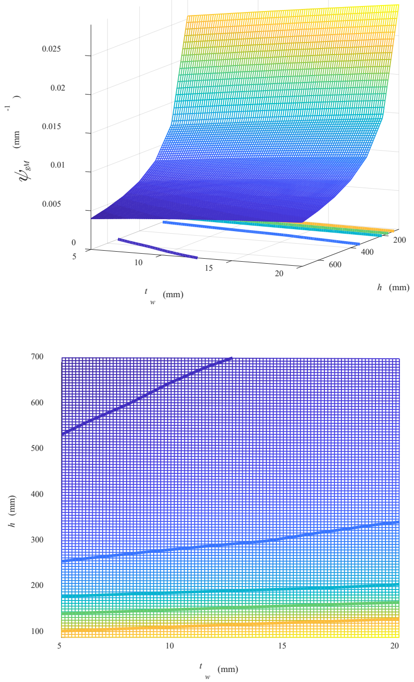

$b = 300, \quad t = 9$

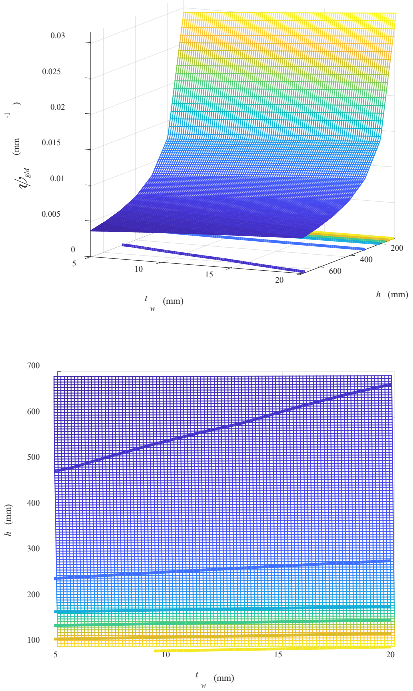

$b = 300, \quad t = 17$

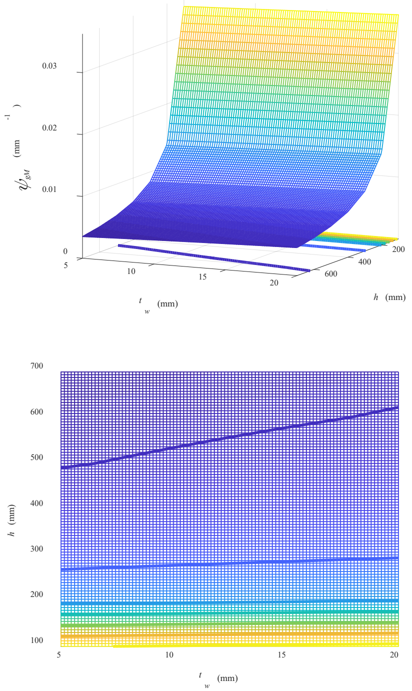

$b = 300, \quad t = 25$

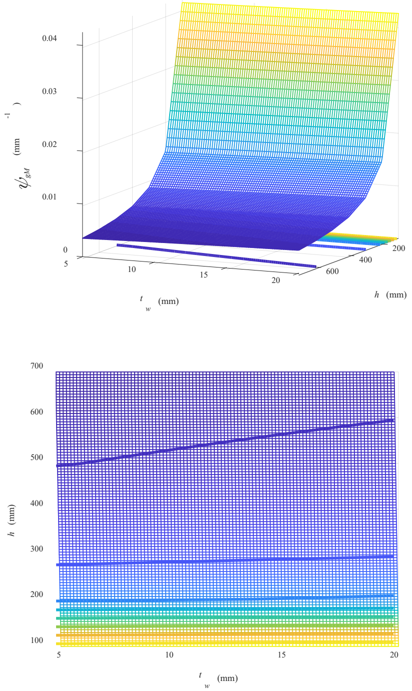

$b = 300, \quad t = 33$

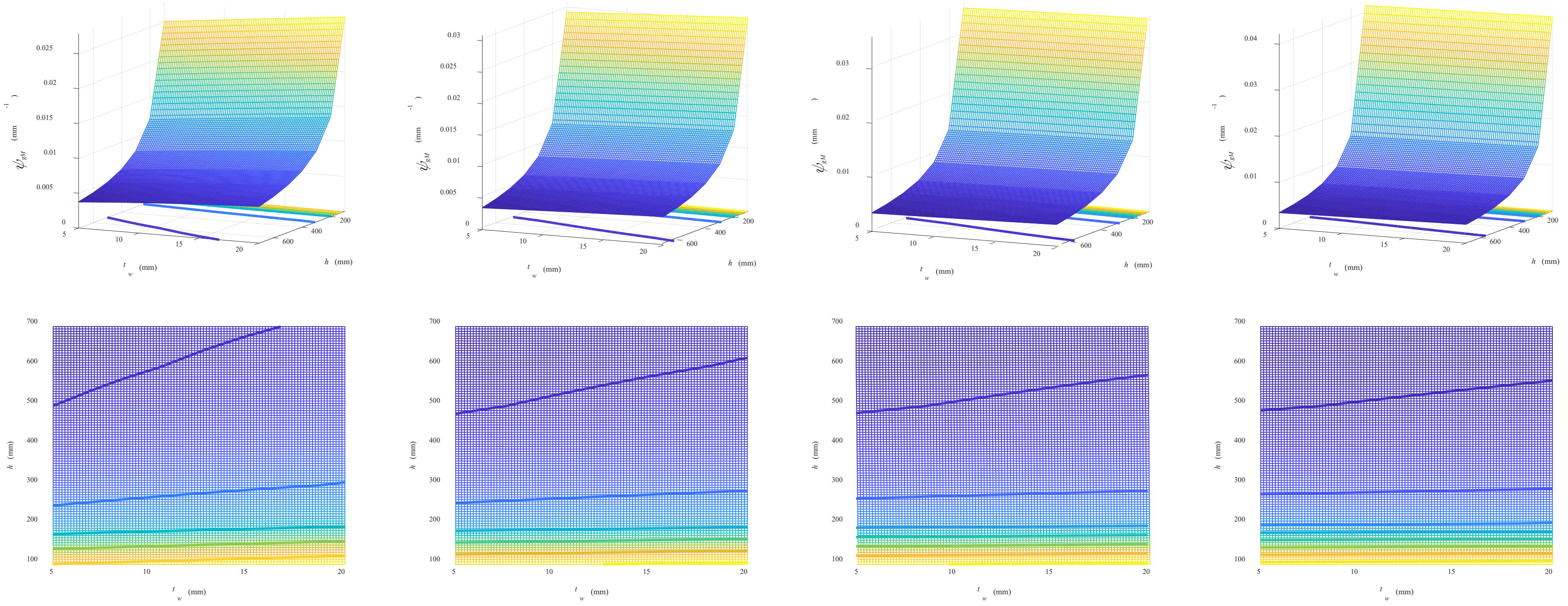

$b = 400$, $t = 9$ $b = 400$, $t = 17$ $b = 400$, $t = 25$ $b = 400$, $t = 33$

Figure S2. Effects of beam height and web thickness on geometry coefficient under different flange width and thickness (mm)

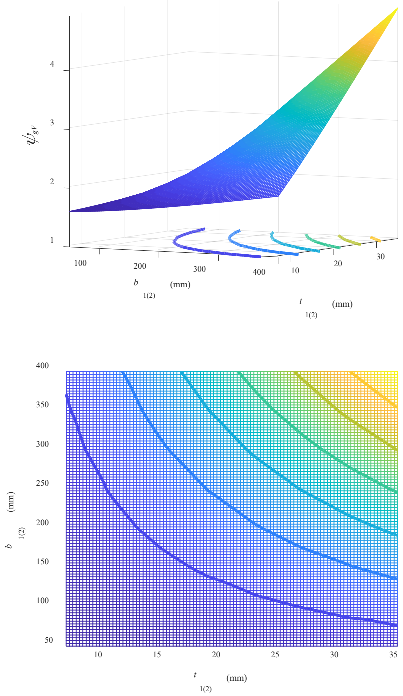

$b_{2(1)} = 100$， $t_{2(1)} = 9$

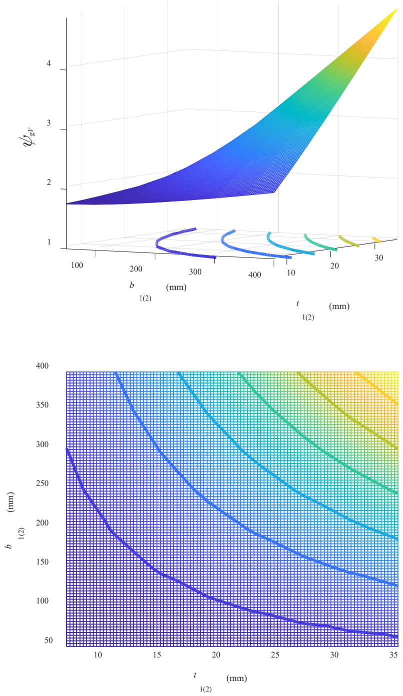

$b_{2(1)} = 100$， $t_{2(1)} = 17$

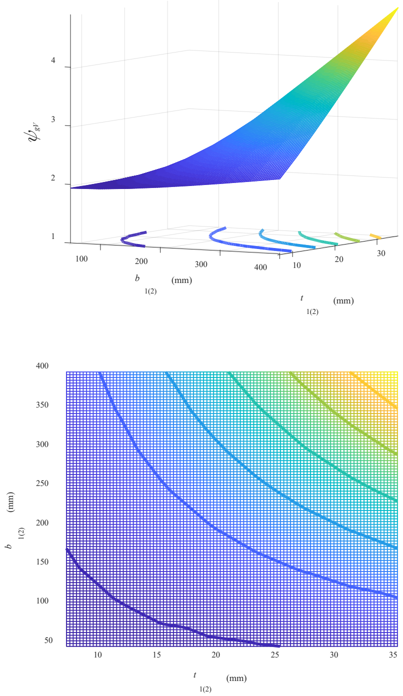

$b_{2(1)} = 100$， $t_{2(1)} = 25$

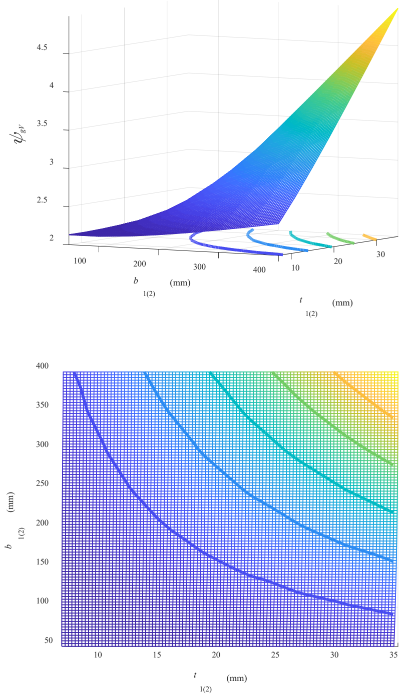

$b_{2(1)} = 100$， $t_{2(1)} = 33$

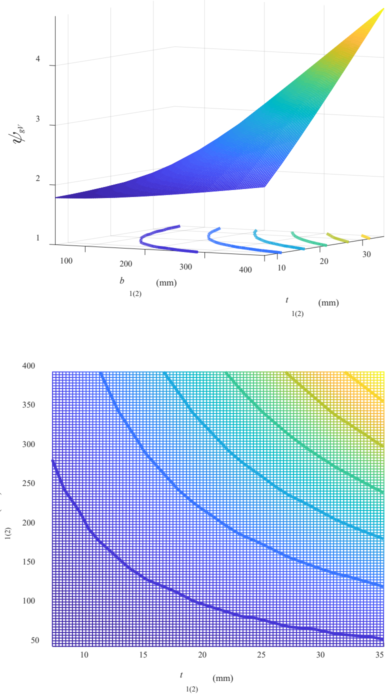

$b_{2(1)}=200,\quad t_{2(1)}=9$

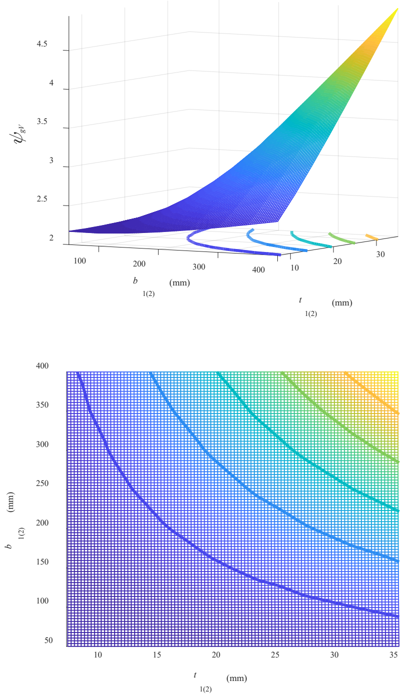

$b_{2(1)}=200,\quad t_{2(1)}=17$

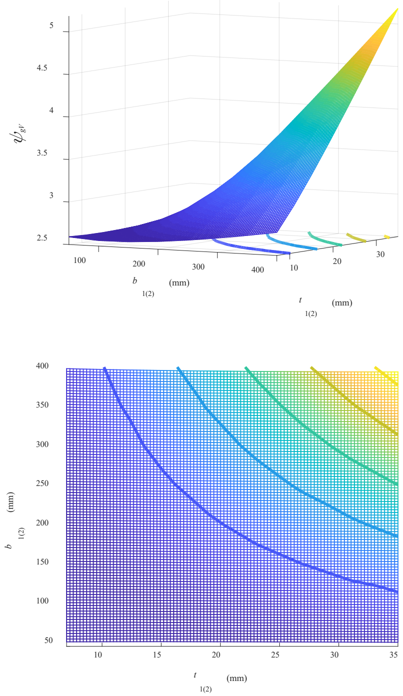

$b_{2(1)}=200,\quad t_{2(1)}=25$

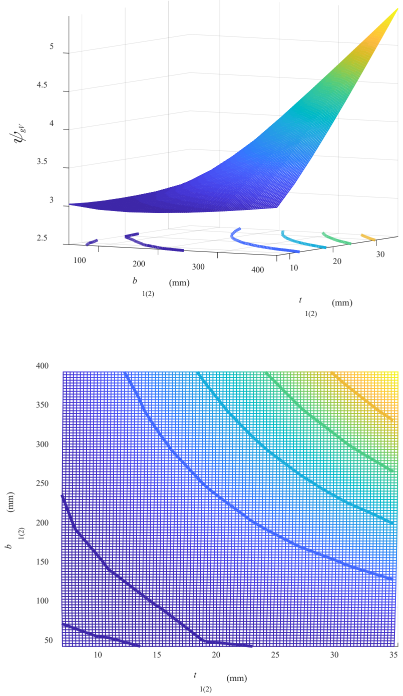

$b_{2(1)}=200,\quad t_{2(1)}=33$

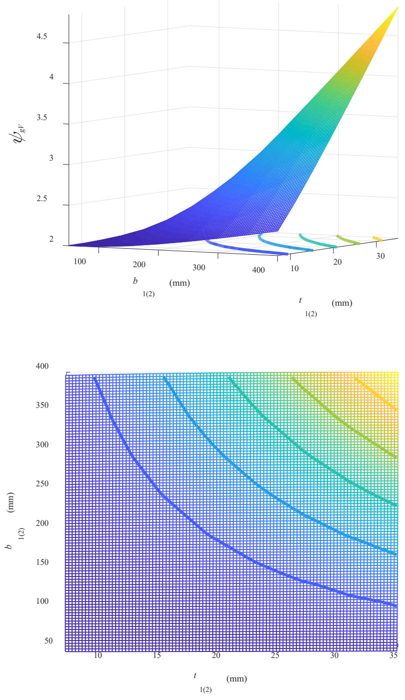

$b_{2(1)} = 300\,,\quad t_{2(1)} = 9$

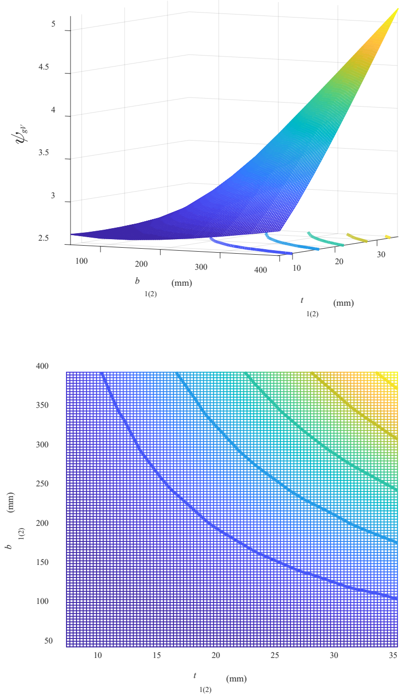

$b_{2(1)} = 300\,,\quad t_{2(1)} = 17$

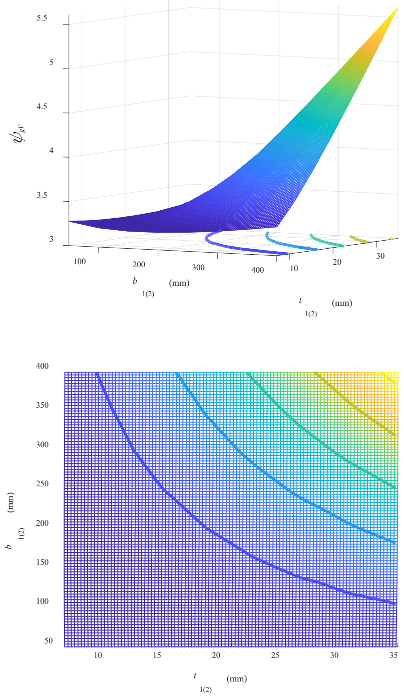

$b_{2(1)} = 300\,,\quad t_{2(1)} = 25$

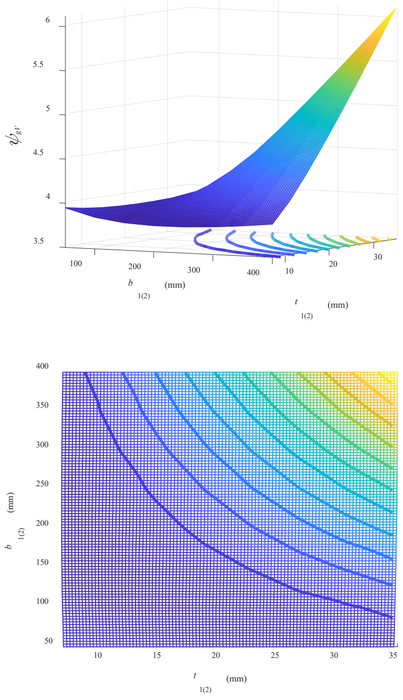

$b_{2(1)} = 300\,,\quad t_{2(1)} = 33$

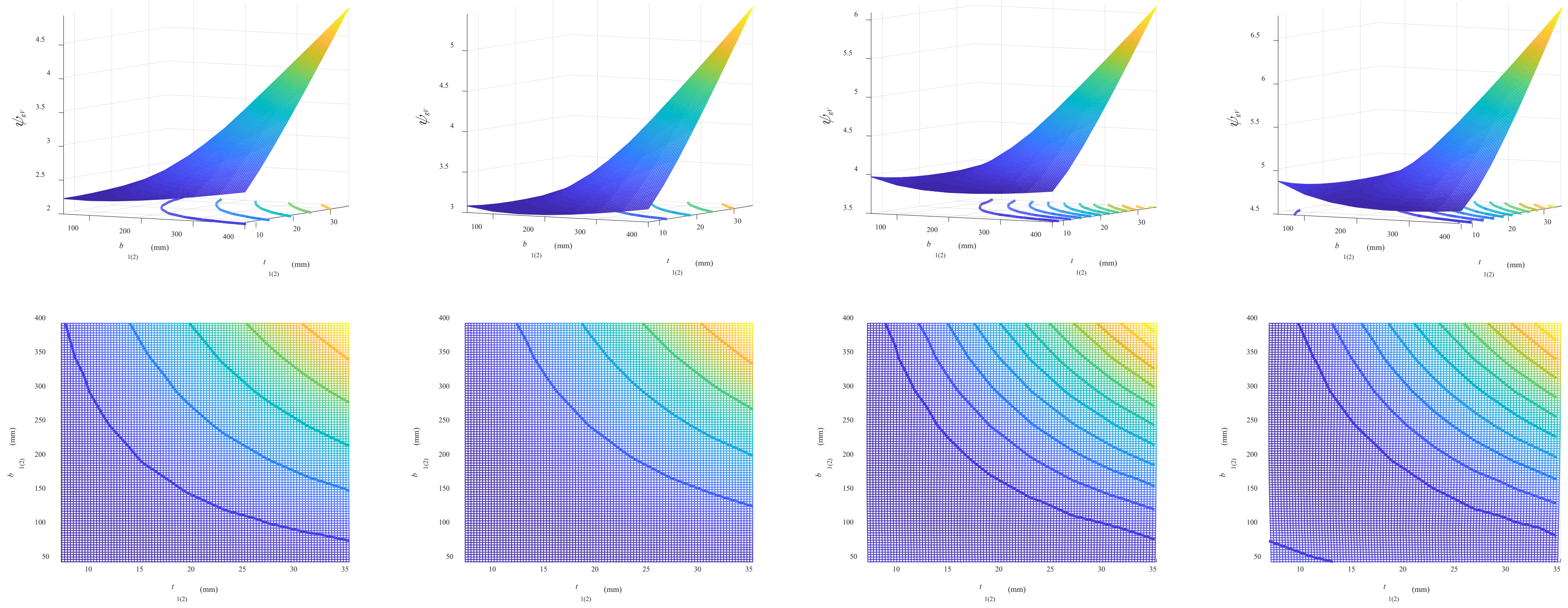

$b_{2(1)} = 400,\ \ t_{2(1)} = 9$ $b_{2(1)} = 400,\ \ t_{2(1)} = 17$ $b_{2(1)} = 400,\ \ t_{2(1)} = 25$ $b_{2(1)} = 400,\ \ t_{2(1)} = 33$

Figure S3. Effects of upper (lower) flange width and thickness on geometry coefficient under different lower (upper) flange width and thickness (mm)

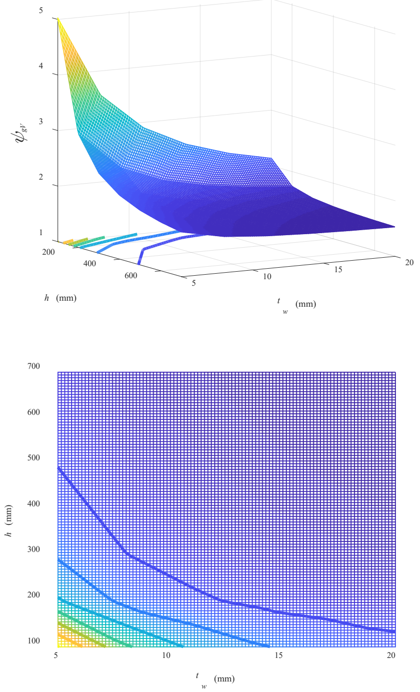

$b = 100$, $t = 9$

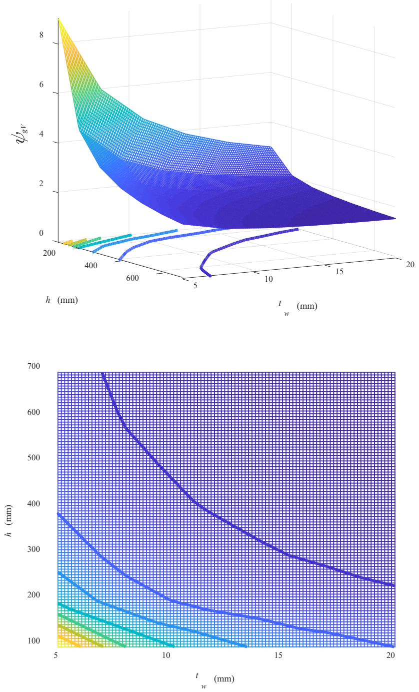

$b = 100$, $t = 17$

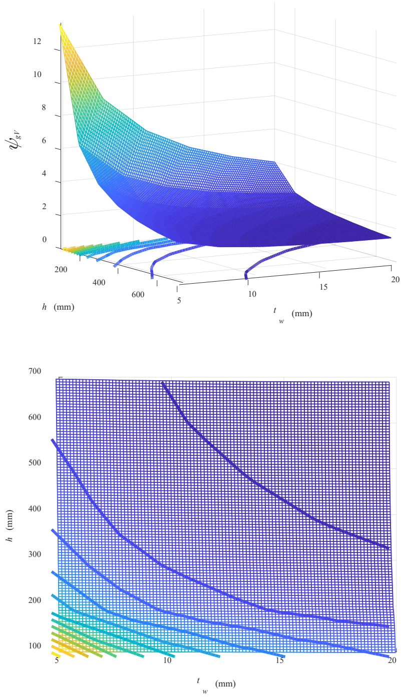

$b = 100$, $t = 25$

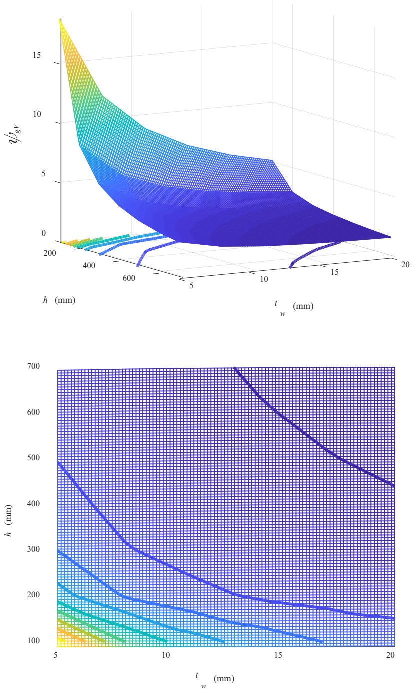

$b = 100$, $t = 33$

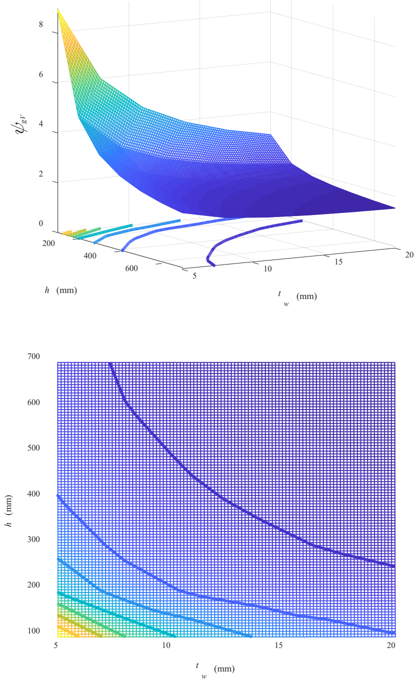

$b = 200\,,\ \ t = 9$

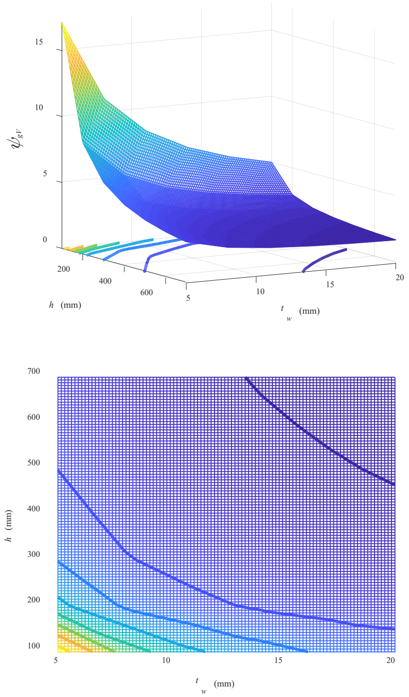

$b = 200\,,\ \ t = 17$

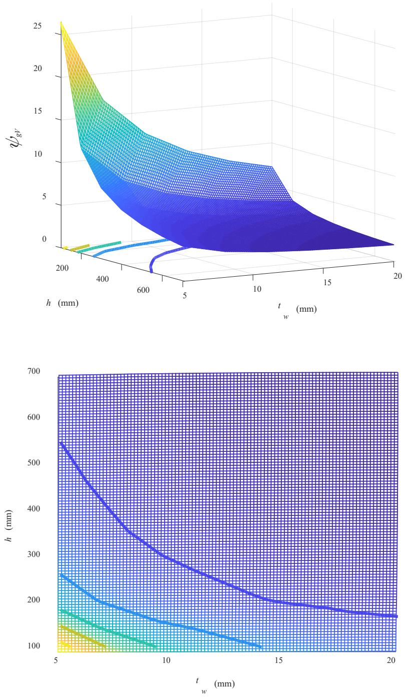

$b = 200\,,\ \ t = 25$

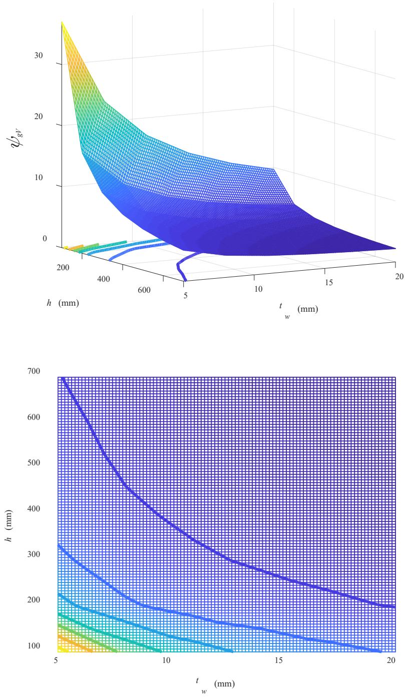

$b = 200\,,\ \ t = 33$

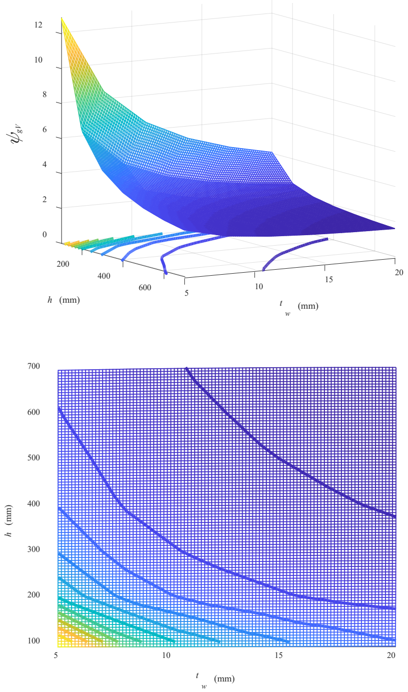

$b = 300$, $t = 9$

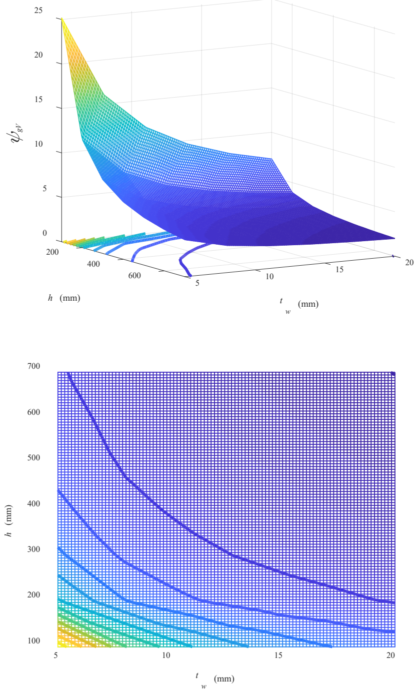

$b = 300$, $t = 17$

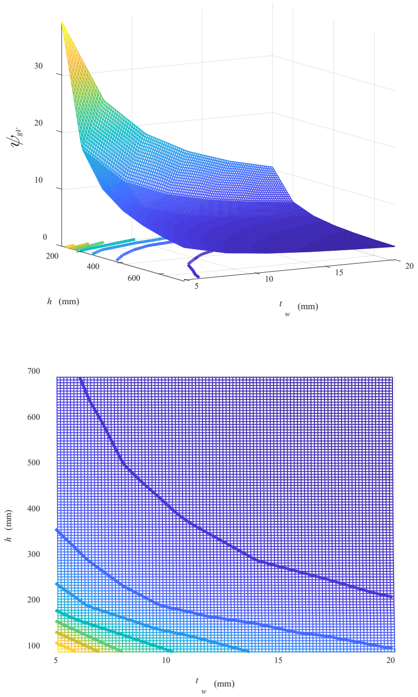

$b = 300$, $t = 25$

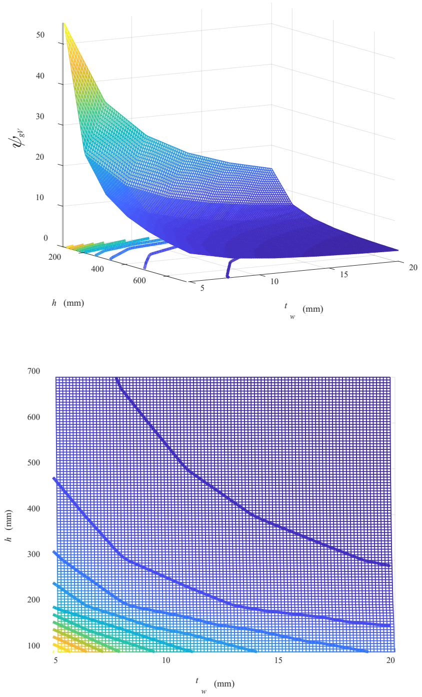

$b = 300$, $t = 33$

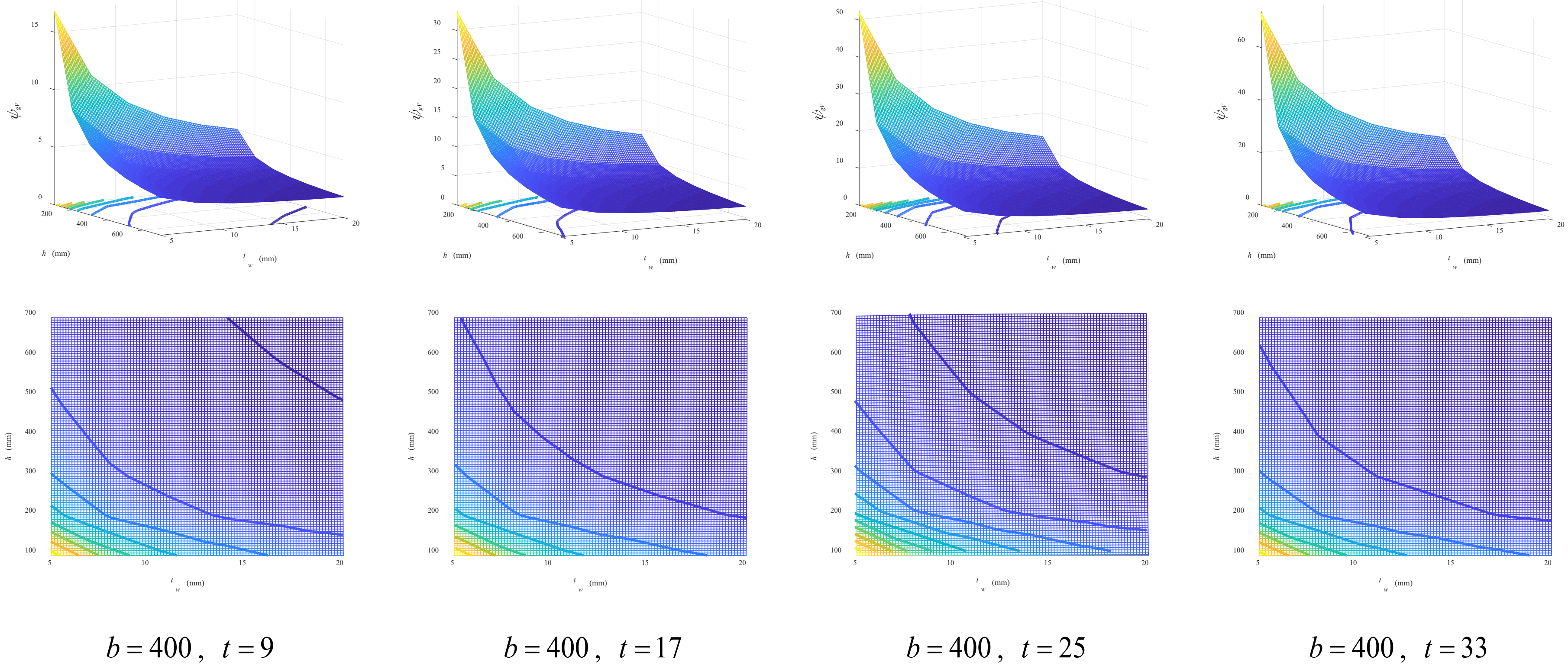

Figure S4. Effects of beam height and web thickness on geometry coefficient under different flange width and thickness (mm)
